# Supplementary material for: SynEM, automated synapse detection for connectomics
Source: eLife. 2017 Jul 14;6:e26414. doi: 10.7554/eLife.26414 (PMC5658066; doi:10.7554/eLife.26414)
Supplement: Supplementary file 1. — Res. Fac: Image voxel volume of SBEM data used in this study relative to the voxel volume in the reported studies. Note that most studies employ data of substantially higher image resolution. DOI: http://dx.doi.org/10.7554/eLife.26414.031 [file elife-26414-supp1.docx]

| **Publication** | **Data** | **Tissue/ Syn type** | **Classification type** | **Feature/ Method** | **Res. Factor** | **Best single syn P_s_/R_s_** | **Comment** |
| --- | --- | --- | --- | --- | --- | --- | --- |
|  |  | **Mammalian cortex** |  |  |  |  |  |
| Mishchenko, Hu, Spacek, Mendenhall, Harris, Chklovskii, 2010 | 2 x 2 x 45 nm^3^ (ssTEM) | Rat CA1 | Boundary voxels | Intensity features orthogonal to boundary | 19.7 | 80%  85% | Fig. 3f |
| Kreshuk, Straehle, Sommer, Koethe, Cantoni, Knott, Hamprecht, 2011 | 5 x 5 x 9 nm^3^ (FIBSEM) | Rat S1 | Voxel | EVs Hess, EVs Structure Tensor, Gaussian smoothing, Gauss Gradient magnitude, Laplacian of Gaussian, difference of Gaussians | 15.7 | 89%  92% | Improved by Becker et al., 2012 (see below). Reported in Fig. 3f |
| **Becker, Ali, Knott, Fua, 2012** | 6.8 x 6.8 x 6.8 nm^3^  5 x 5 x 5 nm^3^  5 x 5 x 5 nm^3^  (FIBSEM) | Rat S1  Rat Hippocampus  Rat Cerebellum | Voxel | Voxel features as in Kreshuk 2011 and context cues | 11.2  28.2  28.2 | 100%  100% | Used for comparison. Fig. 3f, Fig 3 – figure suppl. 3 |
| Kreshuk, Koethe, Pax, Bock, Hamprecht, 2014 | 4.5 x 4.5 x 45 nm^3^ (ssTEM) | Mouse V1 | Voxel & object classification | Voxel based features as in 2011  Object based features (summary statistics over synapse segmentation, local binary pattern, ratio of principal components) | 3.8 | 92.9%  88.2% | Not better in our data than Becker et al., 2012. Reported in Fig. 3f |
| Perez, Seyedhosseini, Deerinck, Bushong, Panda, Tasdizen, Ellisman, 2014 | 3.9 x 3.9 x 30 nm^3^ (SBEM) | Mouse SCN | Voxel | Image filters | 7.8 | - | Inferior performance than Dorkenwald et al., 2017 |
|  |  |  |  |  |  |  |  |
| Roncal, Pekala, Kaynig-Fittkau, Kleissas, Vogelstein, Pfister, Burns, Vogelstein, Chevillet, Hager, 2015 | 3 x 3 x 30 nm^3^ (ATUM) | Mouse S1 | Voxel | RF:  Intensity  Local Binary Pattern  Image Grad. Magn.  Vesicles  Structure Tensor  CNN | 3.3 | 74%  88%  92%  74% | Used for comparison on ATUM data (Kasthuri et al., 2015),  Fig. 3f and Fig. 3-figure suppl. 4 |
| Neila, Baumela, Gonzalez-Soriano, Rodriguez, DeFelipe, Merchan-Perez, 2016 | 14.7 x 14.7 x 20 nm^3^ | Rat S | Voxel | Gaussian classifier for voxel prediction and conditional random field for segmentation regularization | 0.8 | - | Performance as Kreshuk et al., 2011 |
| **Dorkenwald, Schubert, Killinger, Urban, Mikula, Svara, Kornfeld, 2017** | 9 x 9 x 20 nm^3^  9 x 9 x 21 nm^3^  10 x 10 x 30 nm^3^ | Zebra finch  Zebrafish  Mouse striatum | Voxel | CNN | 2.2  2.1  1.2 | 90%  91%  91%  81%  95%  78% | Used for comparison.  Reported in Fig. 3f and Fig. 3-figure suppl. 3 |
|  |  | **Other neuropil** |  |  |  |  |  |
| Jagadeesh, Anderson, Jones, Marc, Fisher, Manjunath, 2013 | 2.18 x 2.18 x 70 nm^3^  (ATEM) | Rabbit Retina,  Ribbon synapses | Region based | Laplacian of Gaussian (resolution pyramid)  Second order Gaussian derivative (for several angles)  Stability region and shape | 10.6 | - |  |
| Kreshuk, Funke, Cardona, Hamprecht, 2015 | 4.7 x 4.7 x 50 nm^3^ (ssTEM) | Drosophila larva | Voxel and  segment based | Partner detection, graphical model | 3.2 | 78%  90% |  |
| Huang, Scheffer, Plaza, 2016 | 10 x 10 x 10 nm^3^ (FIBSEM) | Drosophila optic lobe (medulla) | Voxel and interface classification | T-bar prediction as in Huang et al. 2014, postsynaptic partner prediction using segment interfaces | 3.5 | - |  |
