# Supplementary material for: SynEM, automated synapse detection for connectomics
Source: eLife. 2017 Jul 14;6:e26414. doi: 10.7554/eLife.26414 (PMC5658066; doi:10.7554/eLife.26414)
Supplement: Supplementary file 2. — These distributions were used in Figure 5 for prediction of connectome precision and recall. DOI: http://dx.doi.org/10.7554/eLife.26414.032 [file elife-26414-supp2.docx]

| **Publication** | **Source/target neurons** | **Neuron pair count (synapses between pair)** |
| --- | --- | --- |
| Feldmeyer et al., 1999 | L4 excitatory /L4 excitatory | 2(2), 5(3), 2(4), 2(5) |
| Feldmeyer et al., 2002 | L4 excitatory / L2/3 pyr. cell | 6(4), 7(5) |
| Feldmeyer et al., 2006 | L2/3 pyr. Cell / L2/3 pyr. cell | 2(2), 5(3), 1(4) |
| Frick et al., 2008 | L5A /L5A | 1(1), 3 (3), 1(5), 1(6) |
| Markram et al., 1997 | L5B /L5B | 2(4), 9(5), 4(6), 3(7), 1(8) |
| - | combined | 1(1), 4(2), 13(3), 11(4), 19(5), 5(6), 3(7), 1(8) |
