# Supplementary material for: SynEM, automated synapse detection for connectomics
Source: eLife. 2017 Jul 14;6:e26414. doi: 10.7554/eLife.26414 (PMC5658066; doi:10.7554/eLife.26414)
Supplement: Supplementary file 3. — DOI: http://dx.doi.org/10.7554/eLife.26414.033 [file elife-26414-supp3.docx]

| **Publication** | **Source/target population** | **Average no of synaptic contacts** |
| --- | --- | --- |
| Koelbl et al., 2015 | L4 FS PV -> L4 exc spiny | 3.7 ± 1.3 (range 2 – 6) |
| Hoffmann et al., 2015 | L2/3 inhibitory /L2/3 pyr. cell | 6.2 ± 2 (range 3 – 10) |
| Gupta et al., 2000 | Cortical GABAergic interneurons | F1 9.3 ± 3.1, F2 16 ± 5.5,  F3 16.7 ± 11.9 |
| Markram et al., 2004 | Cortical inh. Interneurons -> pyr. cells | LBC -> PC 14.5 ± 1.7  NBC -> PC 15.8 ± 4.1  SBC -> PC 20.5 ± 10.5  BTC -> PC 15.0 ± 7.1  MC -> PC 11.2 ± 5.5 |
