# Supplementary material for: SynEM, automated synapse detection for connectomics
Source: eLife. 2017 Jul 14;6:e26414. doi: 10.7554/eLife.26414 (PMC5658066; doi:10.7554/eLife.26414)
Supplement: Supplementary file 4. — Document describing the criteria by which synapses in 3D SBEM data were detected by human expert annotators. These criteria are exemplified for synapses from the test set of the SynEM classifier. DOI: http://dx.doi.org/10.7554/eLife.26414.034 [file elife-26414-supp4.pdf]

Supplementary Synapse Gallery (Staffler et al., SynEM):

Synapse detection in large-scale 3D SBEM Data

Introduction

The following document describes the criteria by which synapses in 3D SBEM data were detected by human expert annotators. These criteria are exemplified for synapses from the test set of the SynEM classifier.

In the following the signatures of a typical synapse between a presynaptic bouton and a postsynaptic spine are shown (scale bar: 500 nm):

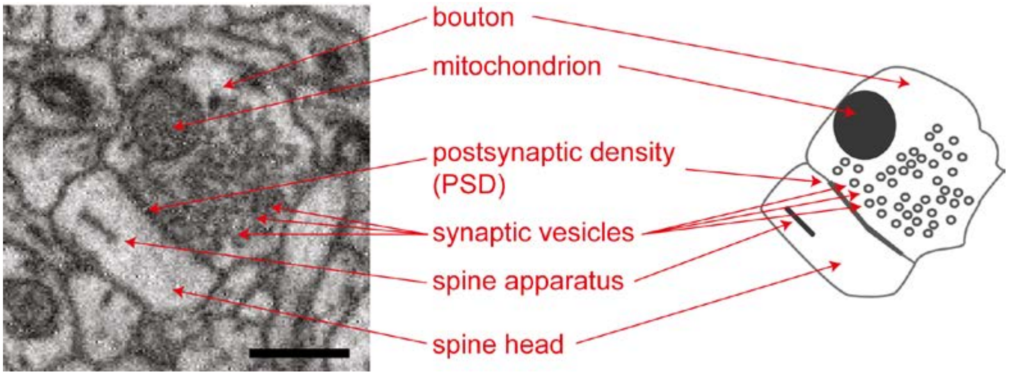

While the single in-plane SBEM image (acquired at a resolution of about 11 nm x 11 nm) does not have the unique clarity of a single high-resolution image obtained by TEM (see for example Colonnier, 1968; Gray, 1959; Harris et al., 1992; Palay, 1956), the 3D nature of SBEM data allows using several (typically at least 10) consecutive images to judge the existence of the synapse:

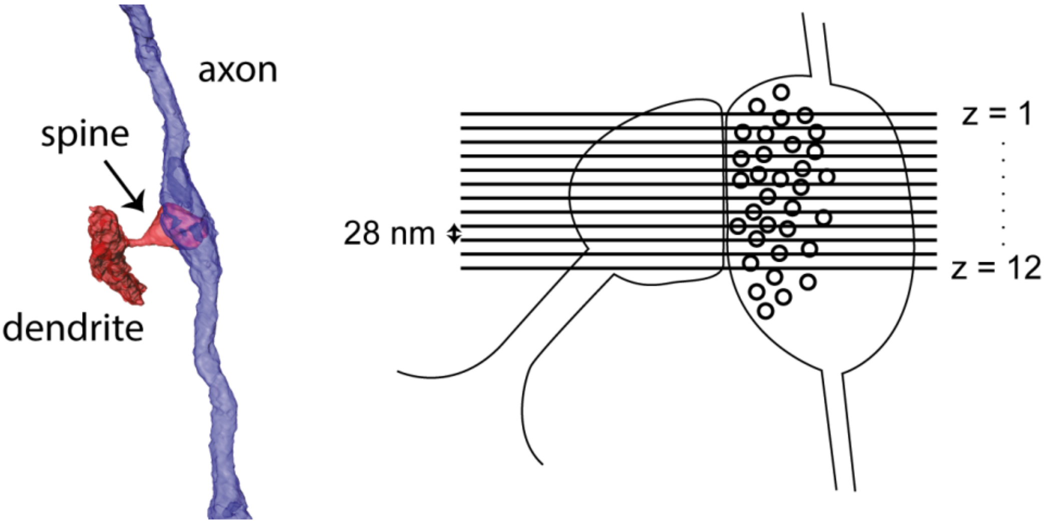

In the following 12 consecutive images from the same synapse are shown:

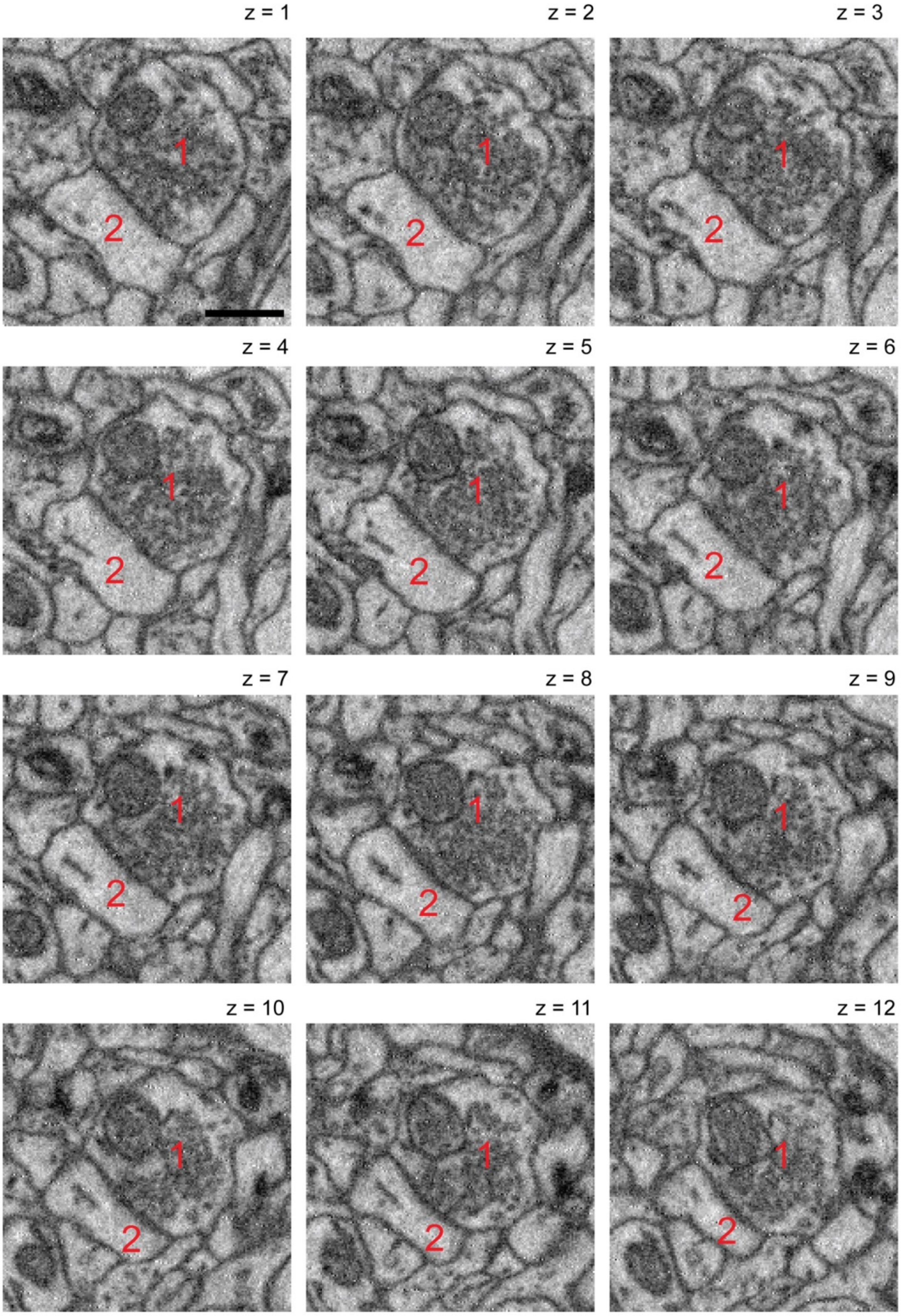

In each of the slices a **vesicle cloud** can be seen (not always single vesicles as e.g. in slice 5 but at least as the entirety of the vesicle accumulation). Furthermore, a **darkening** at the postsynaptic membrane is visible (see e.g. slice 5), and the **vesicle cloud approaches the cell membrane** (see e.g. slices 8 to 11). Finally, the **postsynaptic spine head** including signs of the spine apparatus (slice 6ff.) can be seen. Note that the **vesicle cloud is not attached directly to any other** outer membrane of the presynaptic bouton (see slice 6 ff.).

In the following a second example of a synapse between a synaptic bouton (1) and a postsynaptic spine head (2) can be seen.

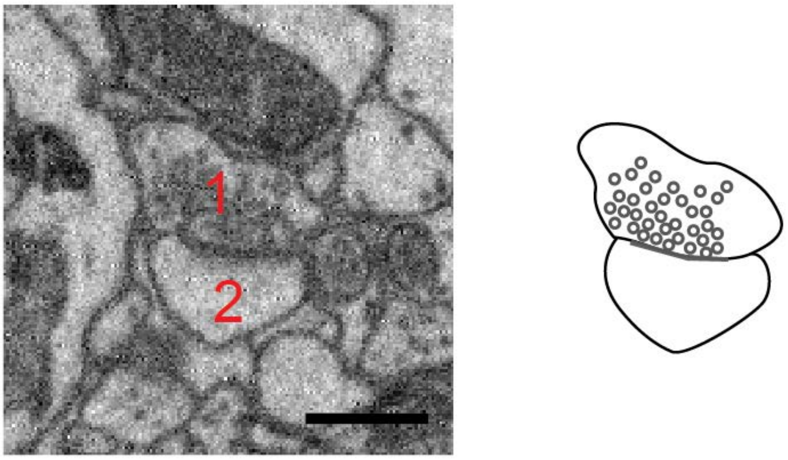

Again, while the single SBEM image may not convey the unique clarity of chemical synapses as in single TEM images, the 3-dimensional image sequence makes the characteristics of a synapse visible as described below:

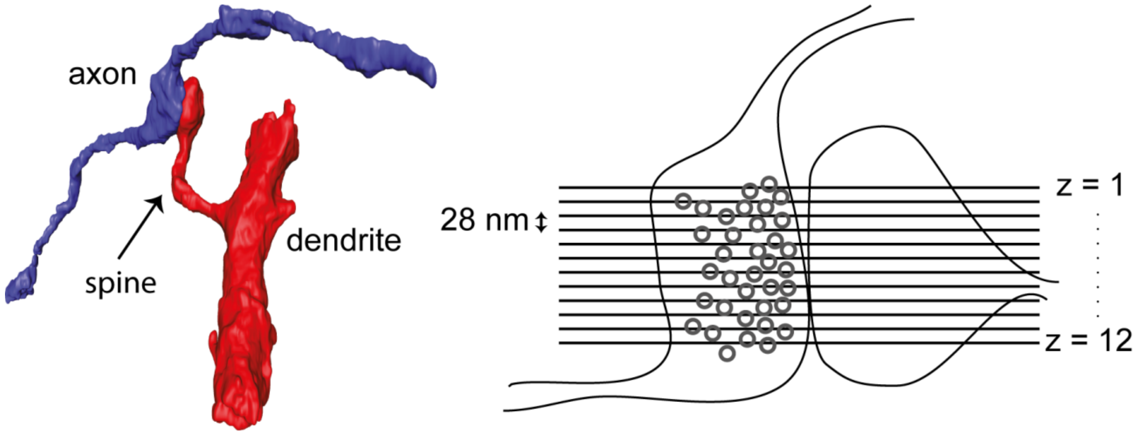

An accumulation of vesicles (slices 1-12) that attach to the likely synaptic membrane (slice 4-7), a darkening of that membrane (slices 6-9) and a postsynaptic spine head

(2) that is not innervated by any other bouton (slices 1-12). (scale bar: 500 nm)

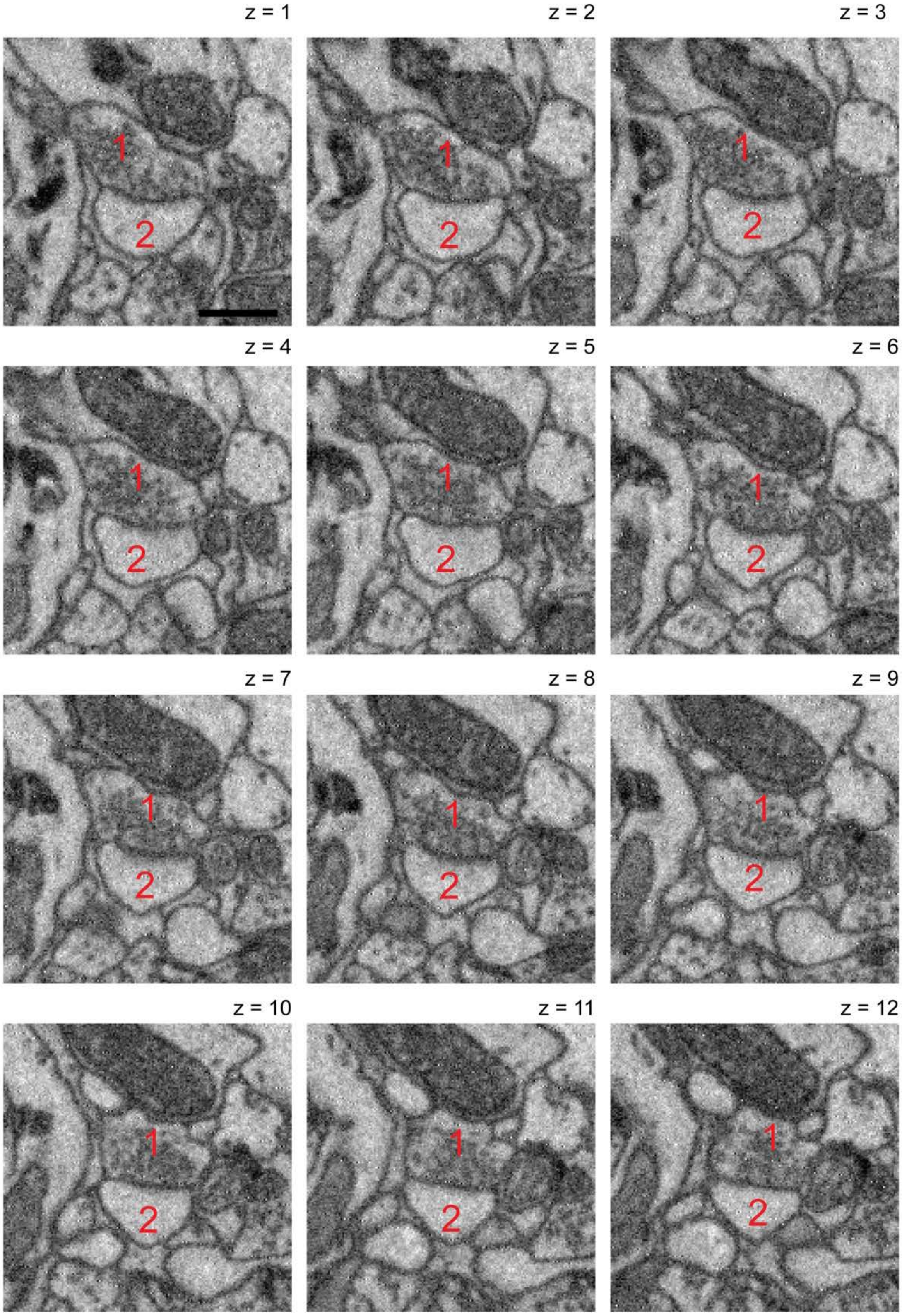

Finally a third example in which two synapses are being made by neighboring boutons (1,3) and spine heads (2,4).

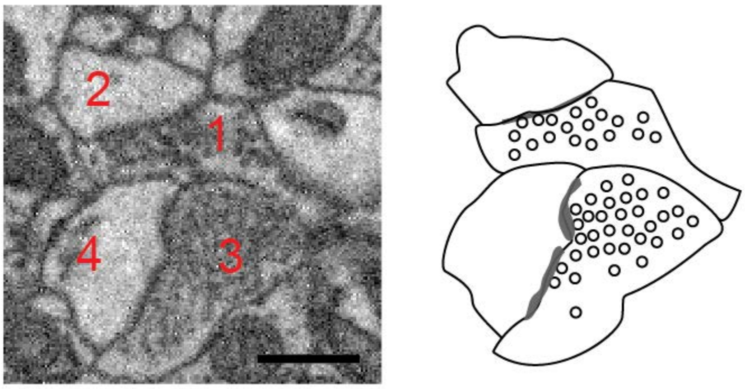

Here, bouton (1) shows a vesicle cloud attached to the synaptic membrane, a slight darkening of that membrane, and a postsynaptic spine head (2). Similar features are visible for bouton (3) and spine head (4). Bouton (1) however does not innervate spine head (4), since the vesicle cloud in (1) approaches spine head (2) but not (4). This makes the existence of a synapse between 1 and 2 and 3 and 4 most likely but not between 1 and 4 (and also not 3 and 2). These criteria are clearer in the full image sequence of planes through that double synaptic configuration as shown below:

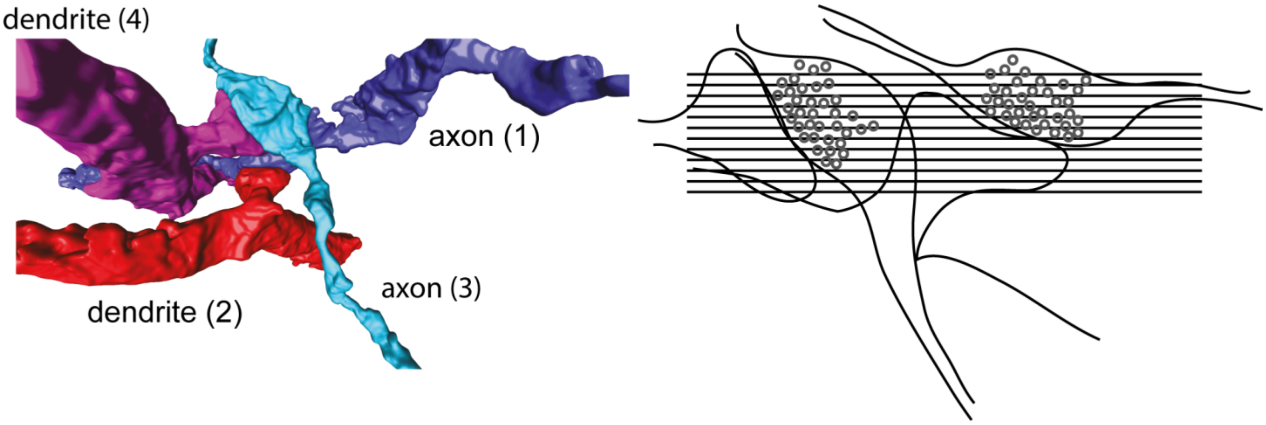

Synapses between bouton (1) and spine head (2) as well as bouton (3) and spine head (4).

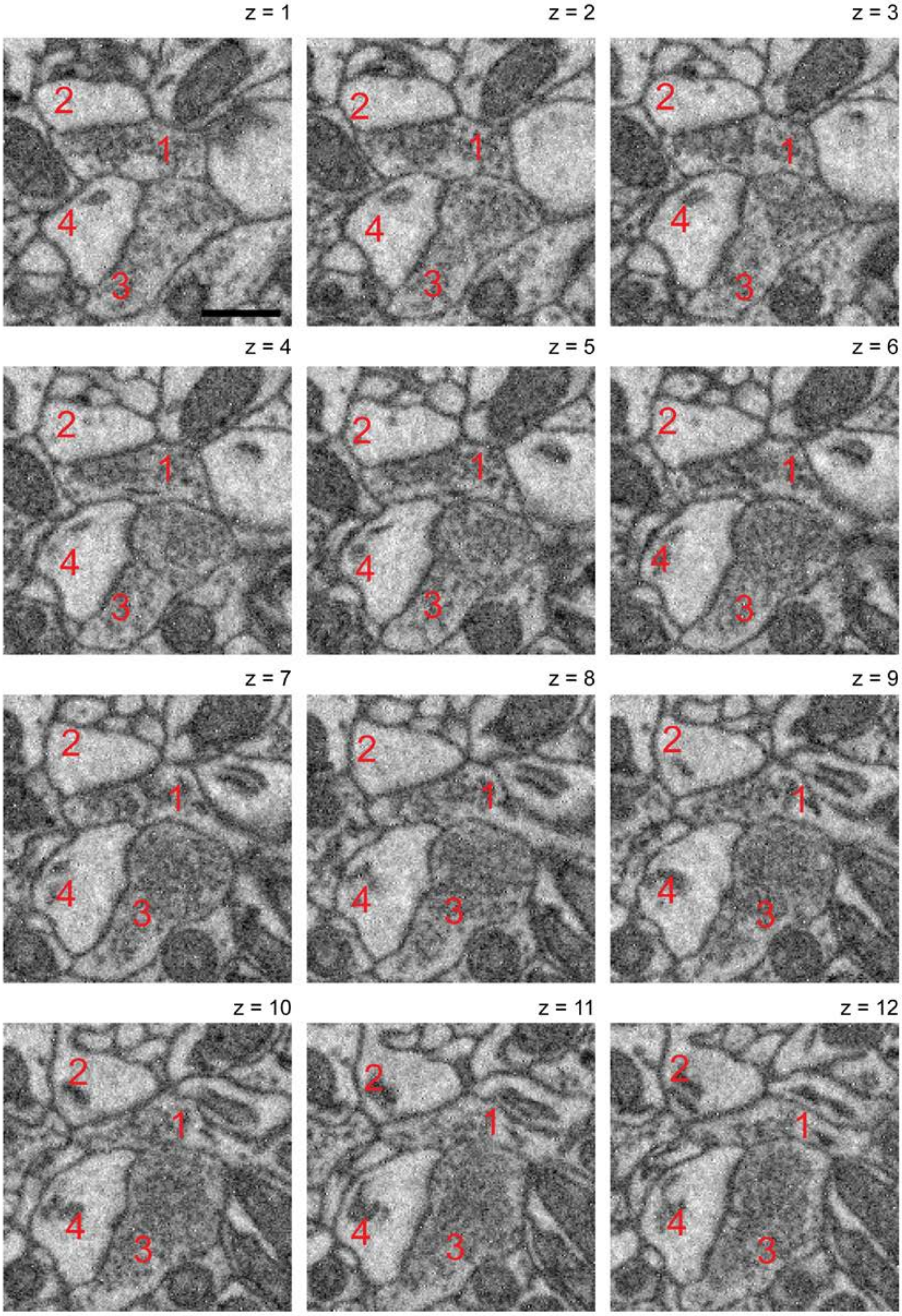

Synapse detection performance by SynEM and human experts

We will next report examples of synapses onto spines in 3D SBEM data from the mouse cortex in relation to the performance of human and automated synapse classification.

Please see first the “confusion table”; i.e. the relationship between human experts’ ground truth interface labels and the SynEM classification results.

		**SynEM**	
		**Synaptic**	**Non-synaptic**
**Ground truth**	**Synaptic**	181 (TP)	23 (FN)
	**Non-synaptic**	11 (FP)	20308 (TN)

When considering the performance of SynEM in relation to synapse size, one finds the following:

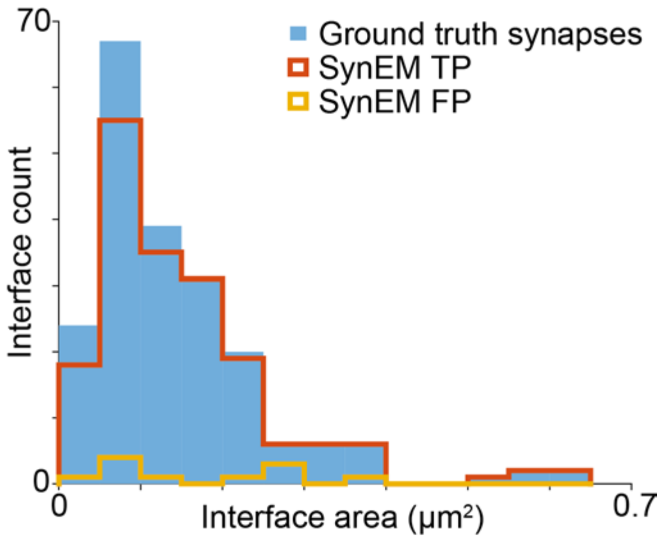

As can be seen, the detection of large synapses is most faithful yielding a recall of 100% for synapses with segmentation interfaces larger than 0.25 µm^2 (cf. SynEM true positive detections (TP), red curve, and ground truth synapses, blue area). For smaller synaptic interfaces the synapse recall decreases to about 95 % in the range between 0.1 and 0.25 µm^2 and drops to 80% for the small fraction of synapses with less than 0.1 µm^2 synaptic interface area. Similarly the false positives are more frequent for smaller synapses.

Interestingly, human expert agreement on synapse detection is also dependent on synapse size similar to SynEM:

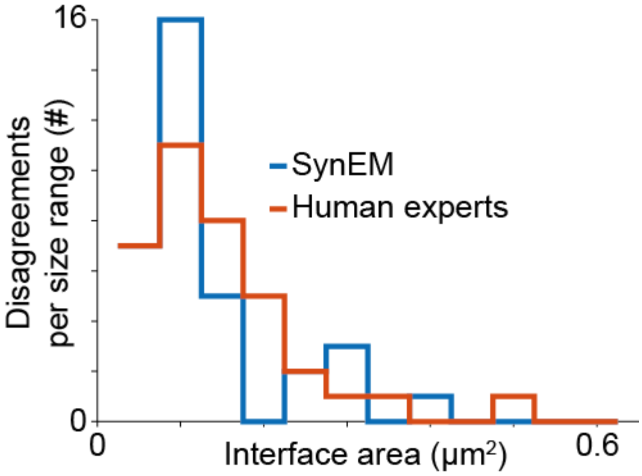

Synapse gallery

To provide representative examples of SynEM synapse detection, we will now report first the 3 largest synapses (TP1-3), which were faithfully detected by SynEM, followed by a random selection of 5 intermediate sized synapses (TP4-8) and then the 3 smallest synapses (TP9-11). Then, we will report 2 random examples of synapses that were missed by SynEM (FN1-2) and finally 2 randomly selected interfaces that were classified as synaptic by SynEM but not by the human experts (FP1-2).

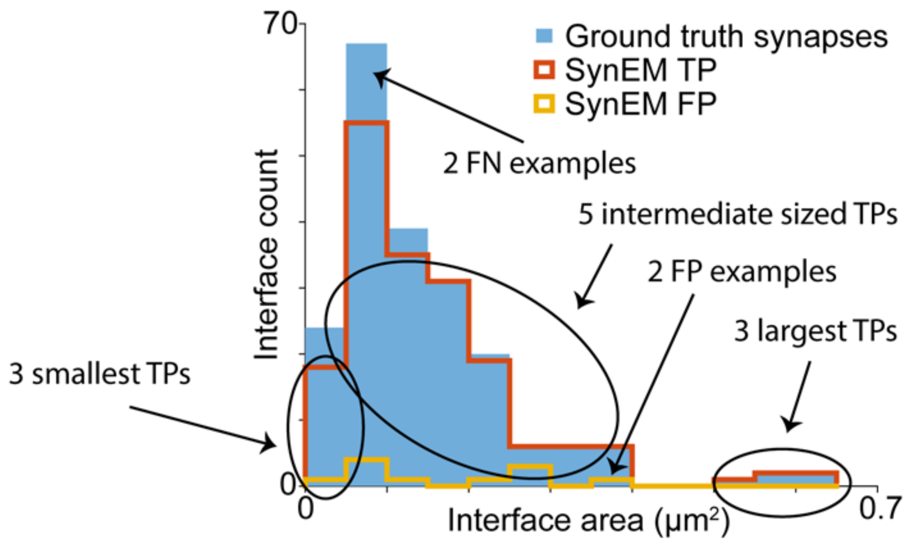

3 largest TP examples

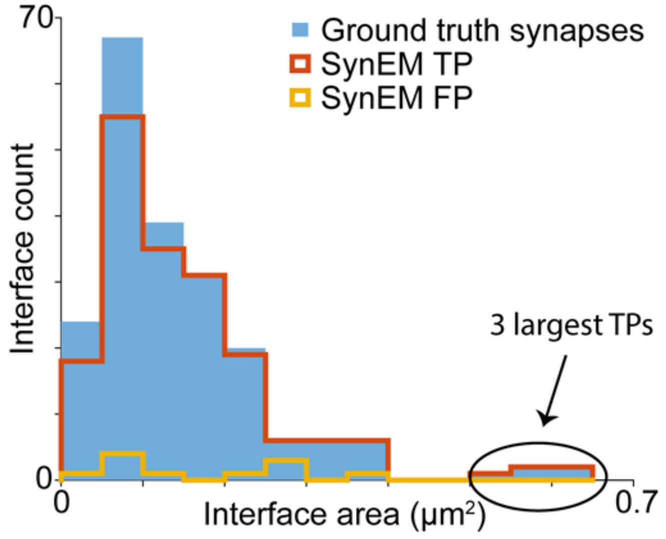

True positive (TP) synapse 01 xy imaging plane

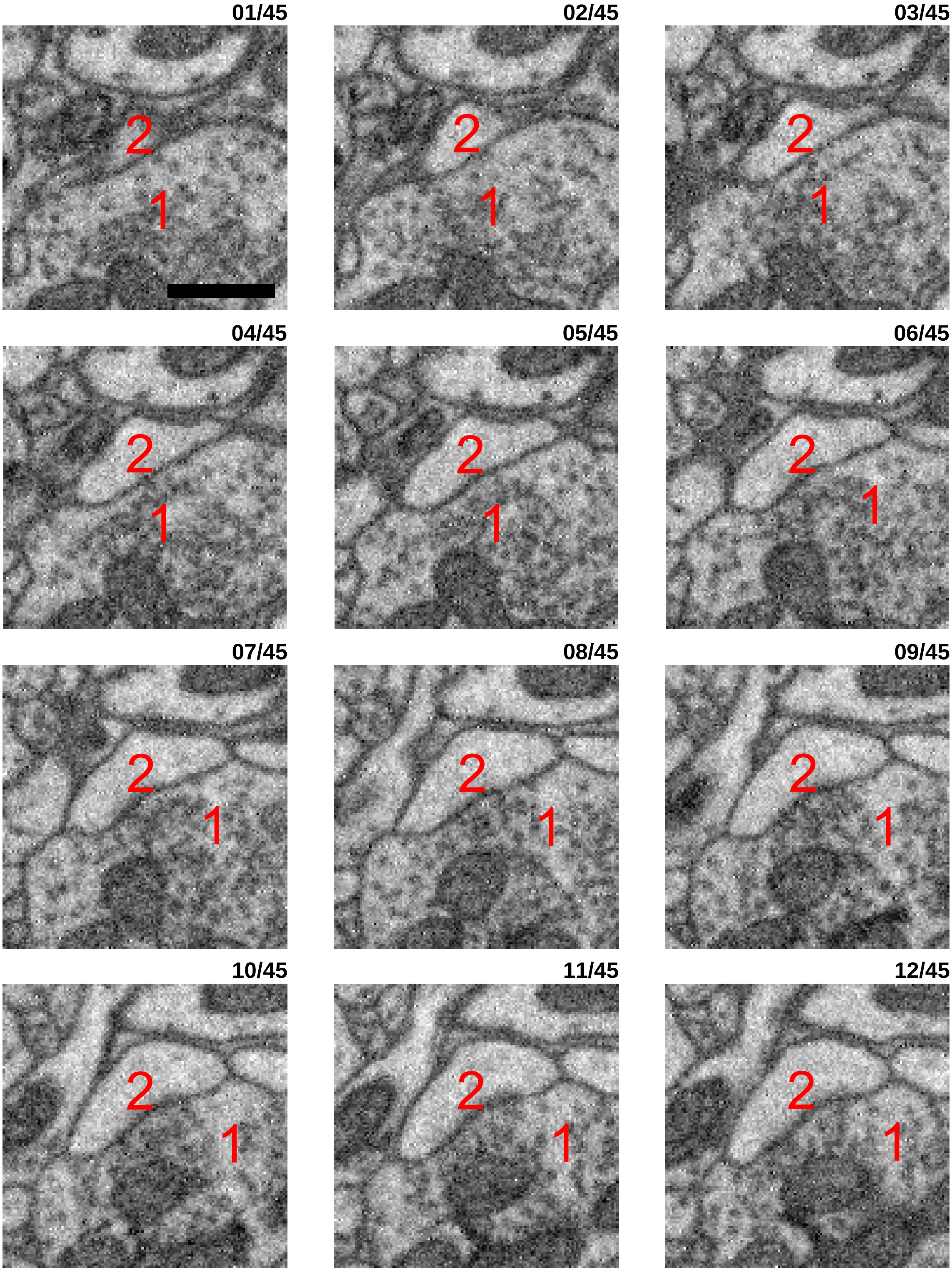

Synapse between large bouton (1) and a spine head (2, slices 8ff): A large presynaptic vesicle cloud (1) that approaches the bouton-to-spine head interface (especially in slices 8 to 11) accompanied by a slight extension and darkening of the postsynaptic membrane in these slices (slices 11 to 15). Note the spine apparatus in the spine head (slices 29ff). Scale bar: 500 nm.

True positive (TP) synapse 01 - cont (2) xy imaging plane

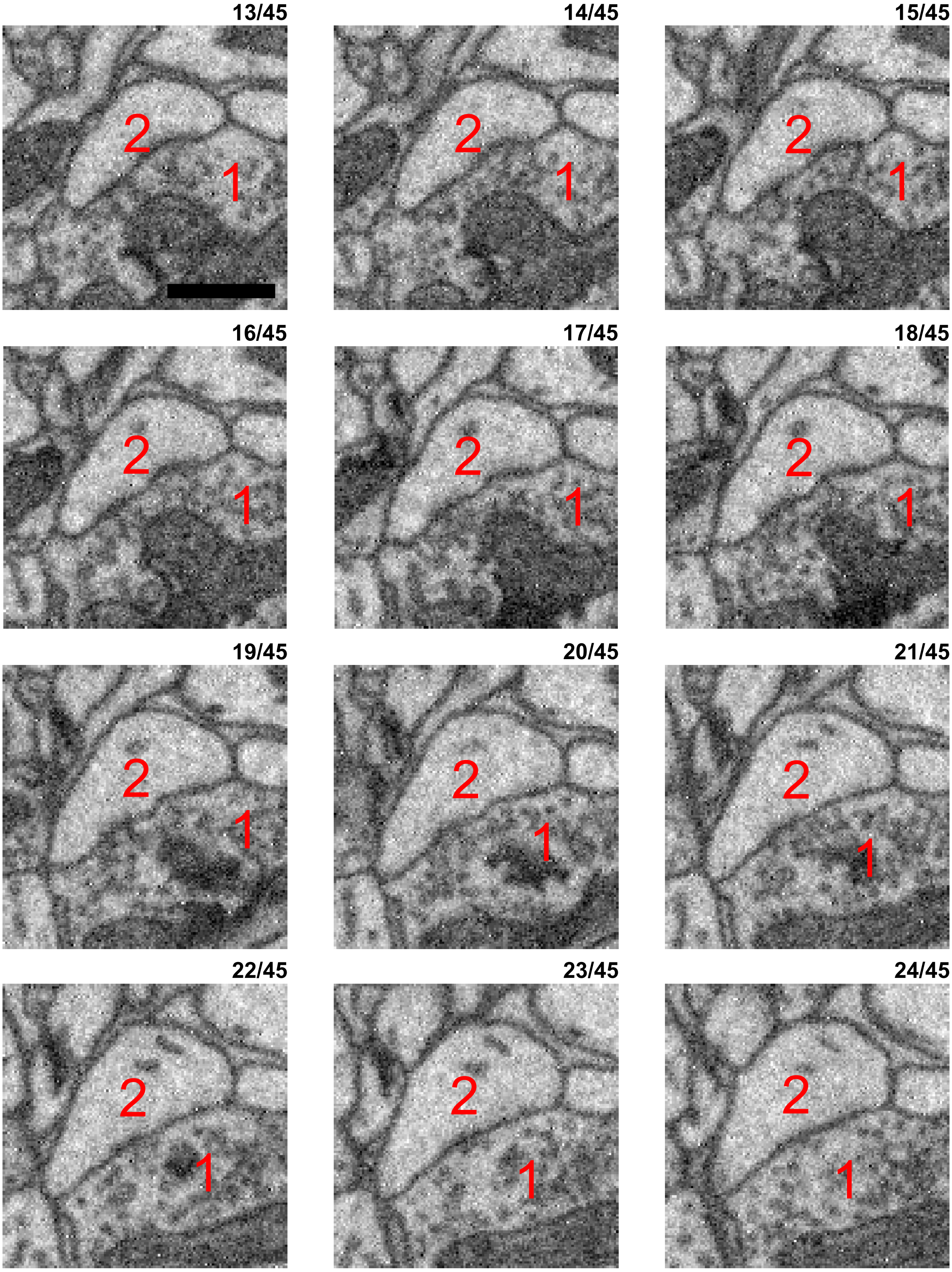

True positive (TP) synapse 01 - cont (3) xy imaging plane

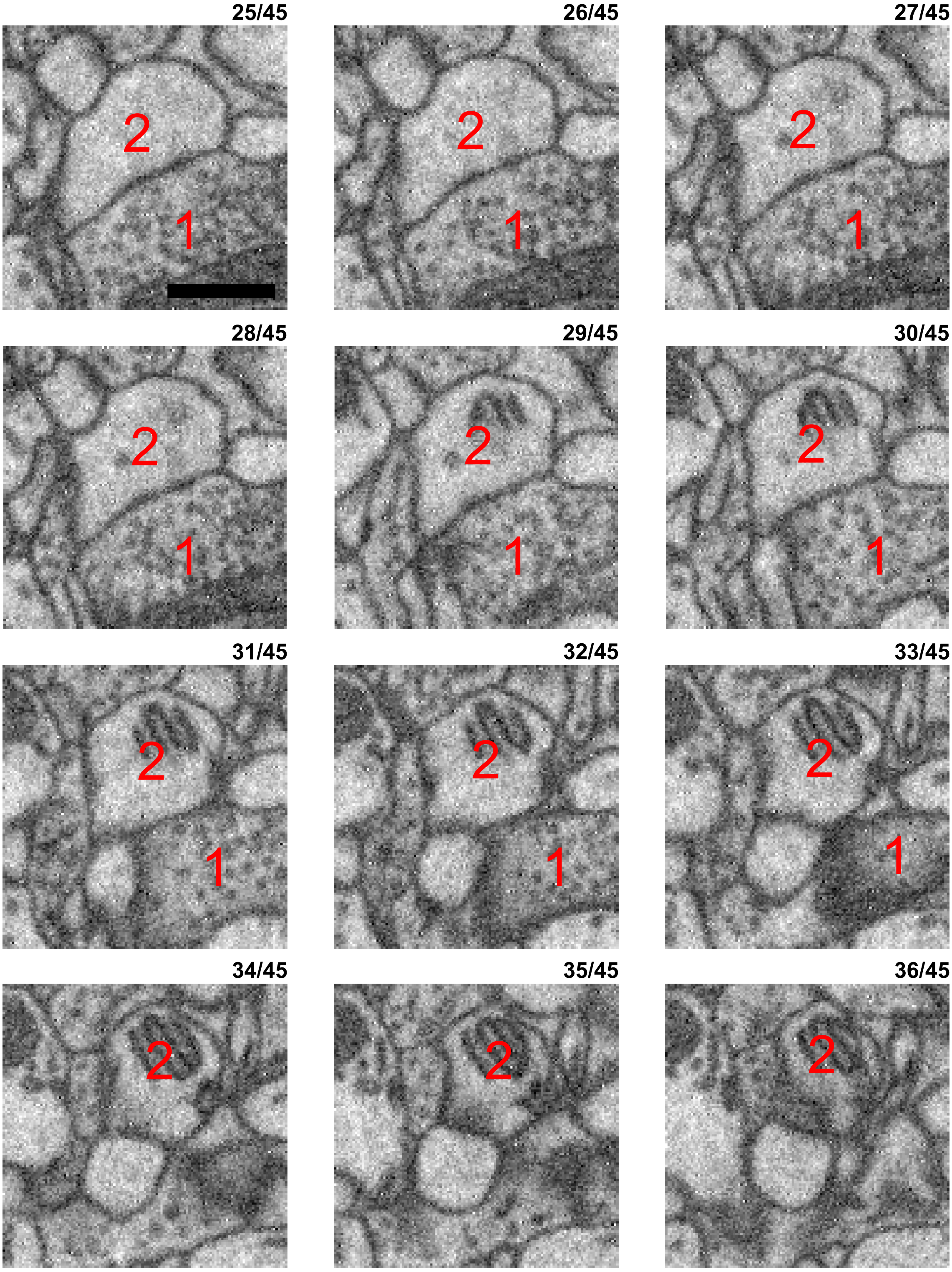

True positive (TP) synapse 01 - cont (4)

xy imaging plane

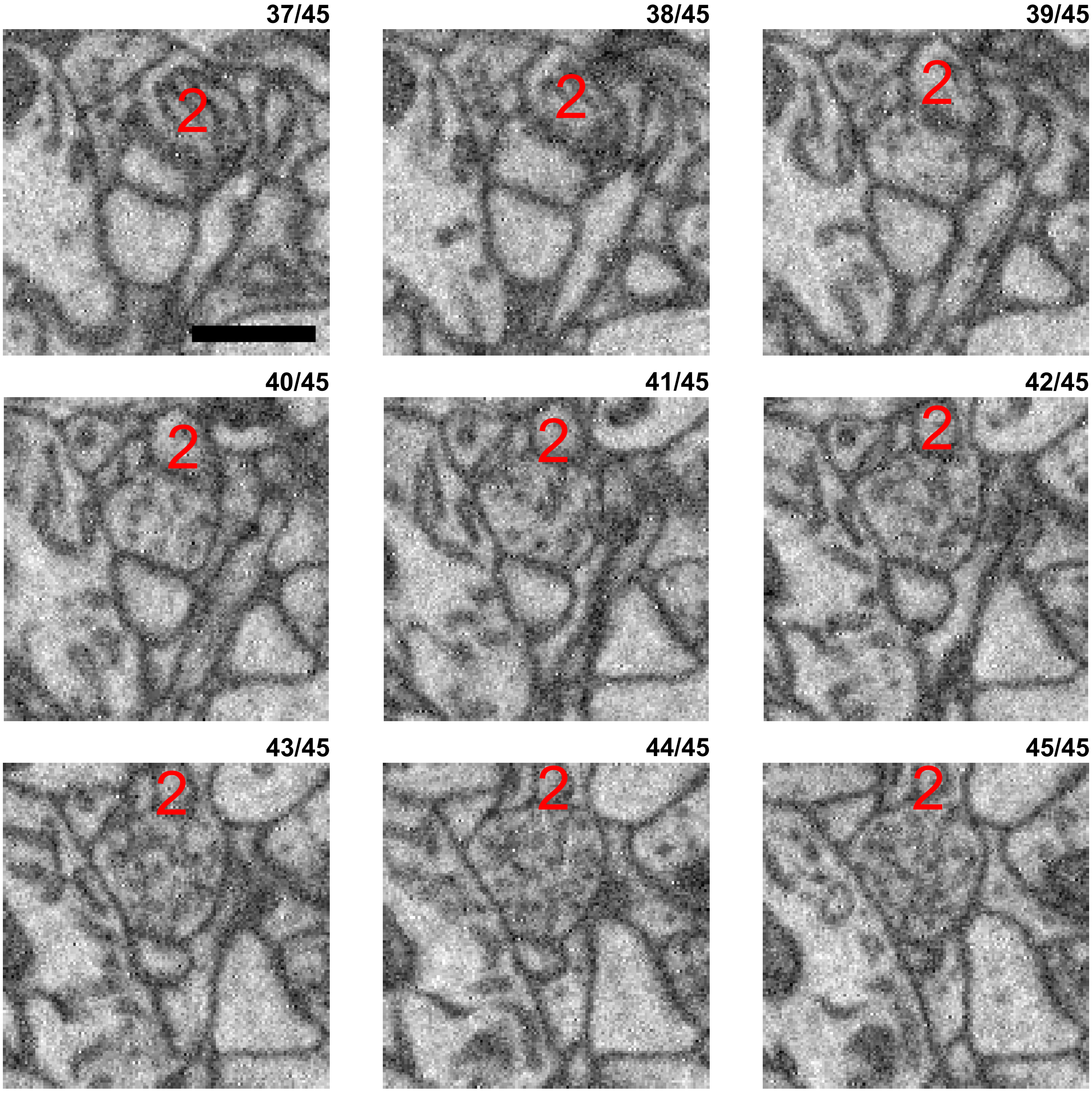

True positive (TP) synapse 02 xy imaging plane

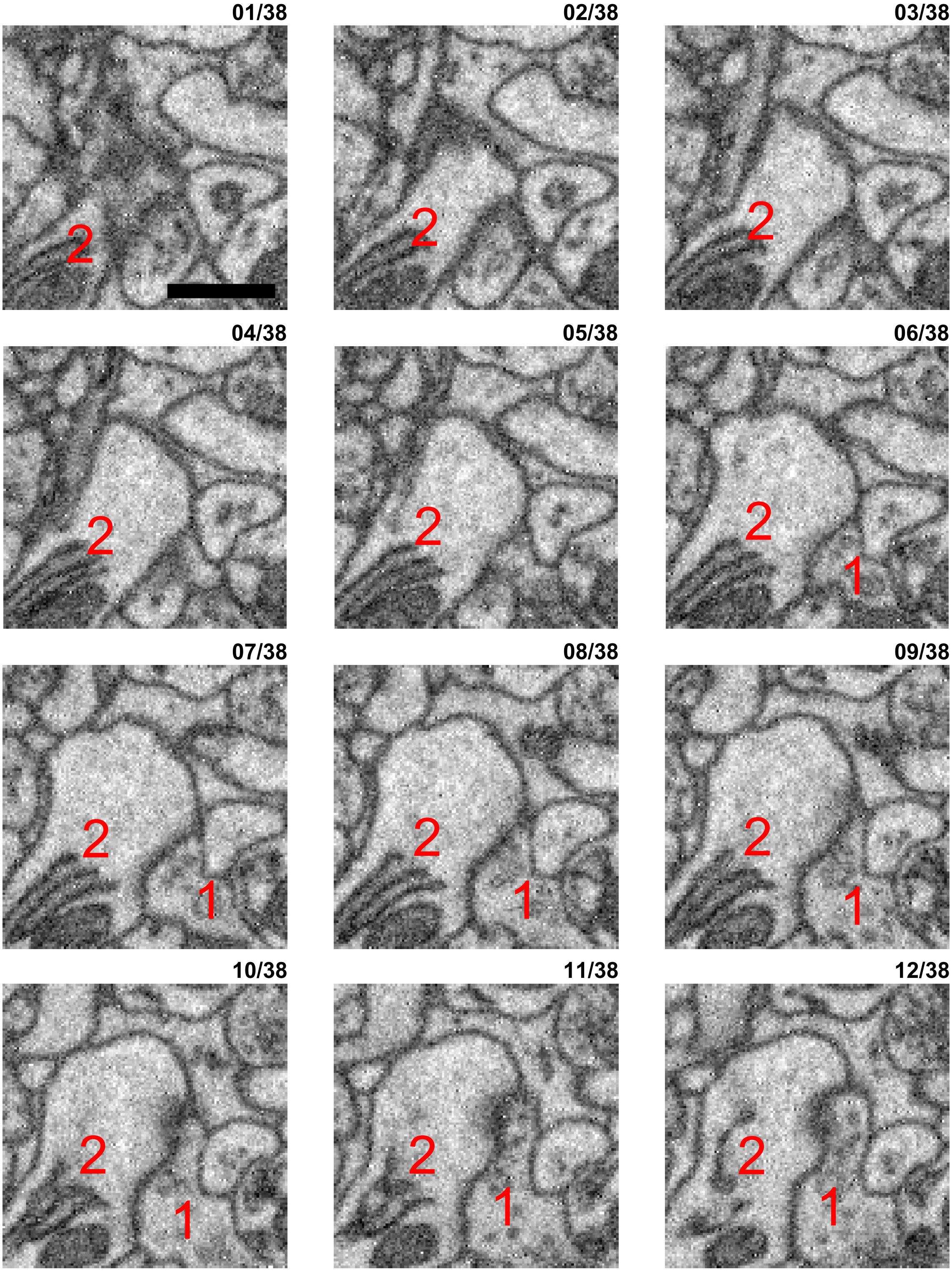

Synapse between an axonal bouton (1, slices 16ff) and a spine head (2, slices 3-30). Medium size presynaptic vesicle cloud (1, slices 20ff) that approaches the candidate synaptic membrane directly in slices 23ff, together with a darkening of the postsynaptic membrane, potentially at two main sites (visible for example in slices 22ff). The postsynaptic spine head is not approached by an alternative innervation and the presynaptic bouton similarly does not have a competing candidate target.

True positive (TP) synapse 02 - cont (2) xy imaging plane

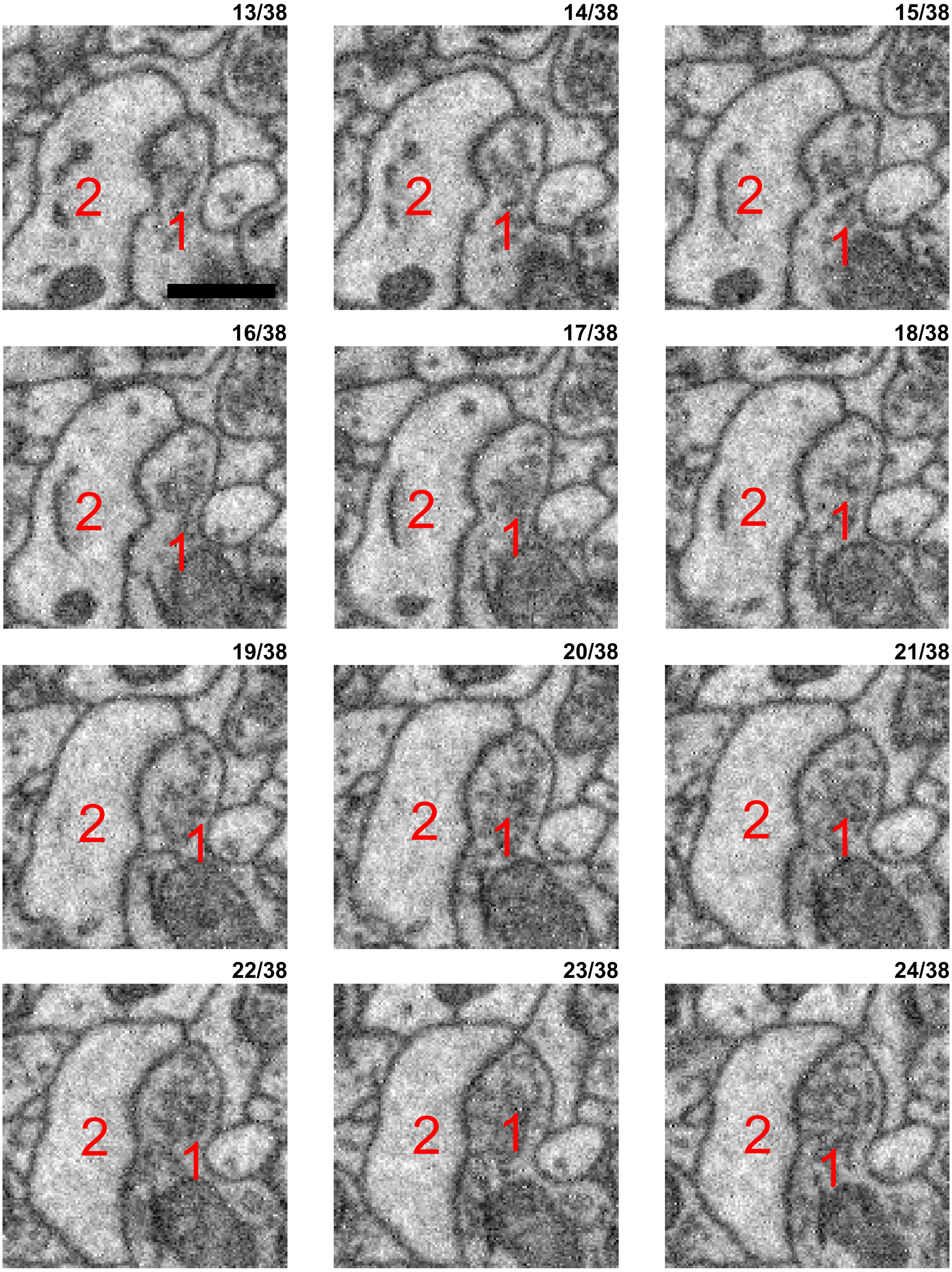

True positive (TP) synapse 02 - cont (3) xy imaging plane

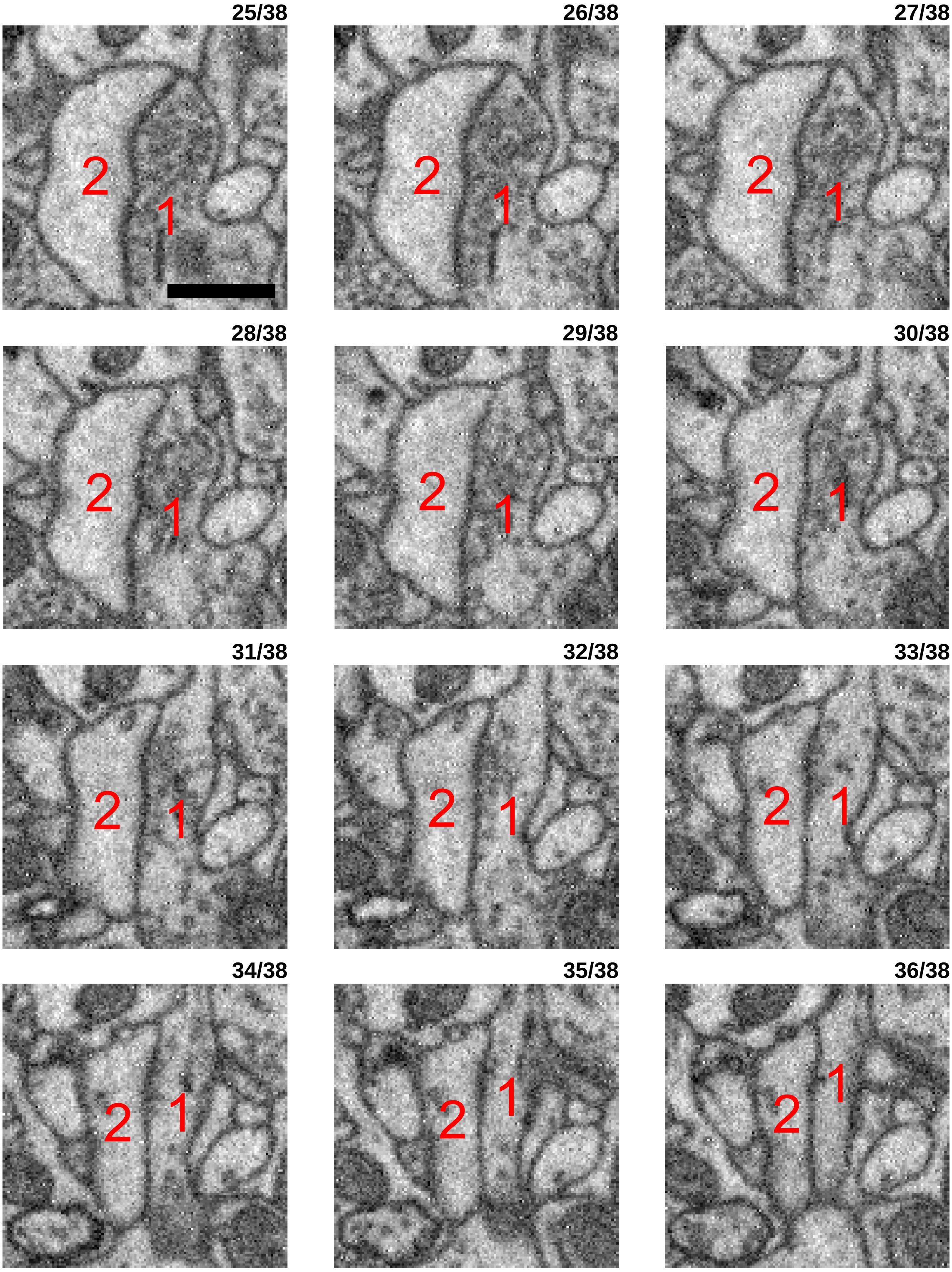

True positive (TP) synapse 02 - cont (4)

xy imaging plane

37/38

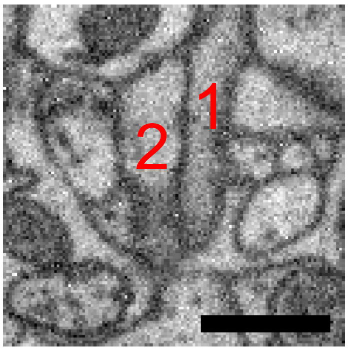

38/38

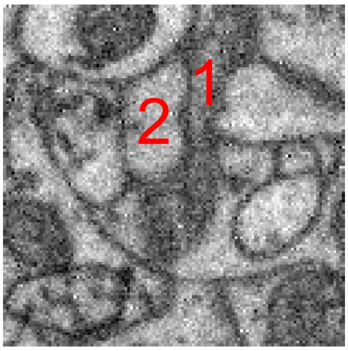

True positive (TP) synapse 03 yz orthogonal reslice

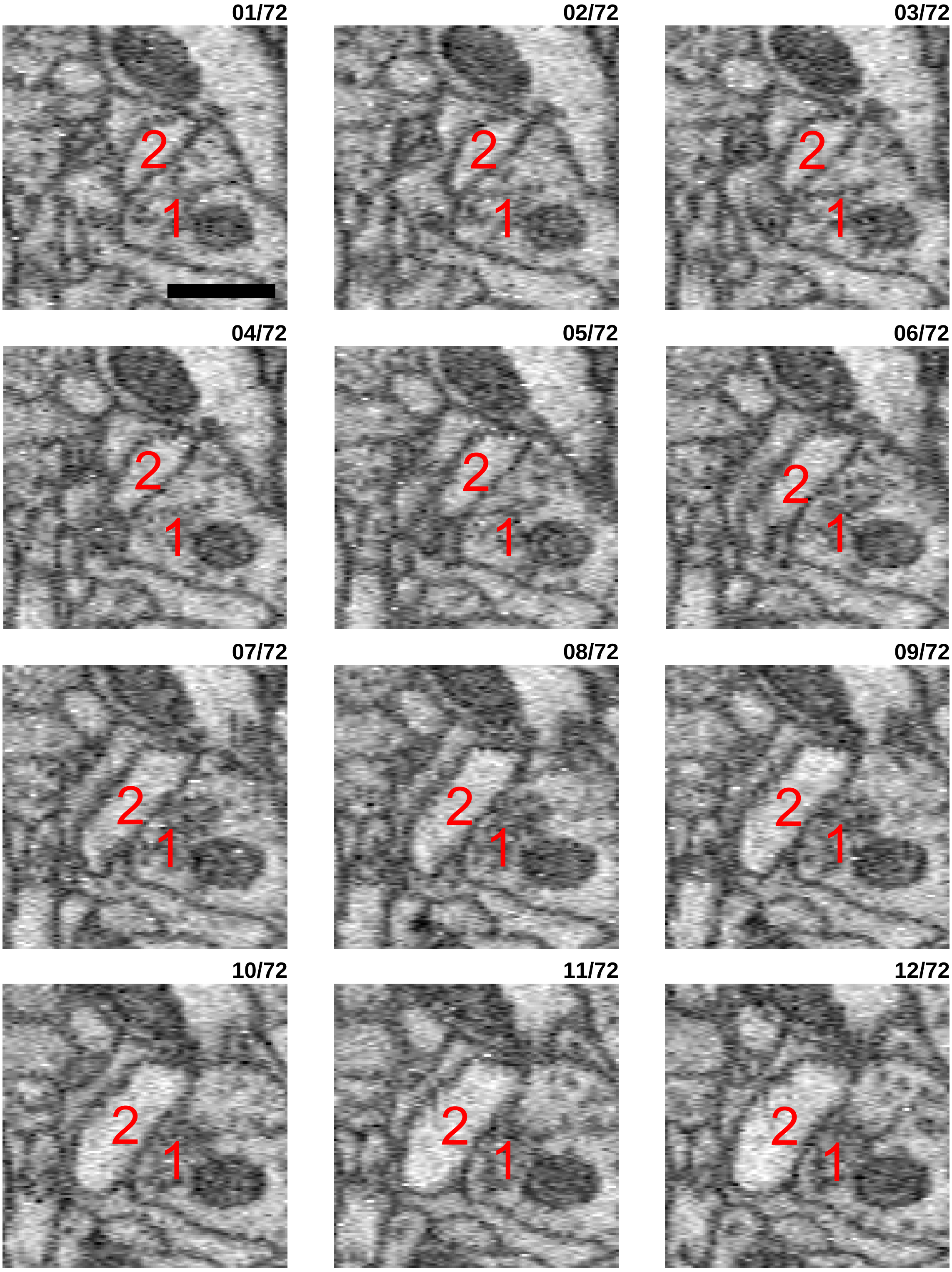

Synapse best visible in one of the orthogonal reslices (yz plane) due to its oblique orientation to the imaging plane. Relatively large vesicle cloud (1, slices 25ff) visible in the synaptic bouton (starting at about slice 25 extending all the way to slice 58 or 60). Vesicle cloud clearly approaches the postsynaptic spine head (e. g. slices 25, 26 and 30ff), with a darkening of the membrane, for example slices 25ff and again slices 35ff. The postsynaptic spine head (2) is not approached by any competing bouton (slices 1 to 72).

True positive (TP) synapse 03 - cont (2) yz orthogonal reslice

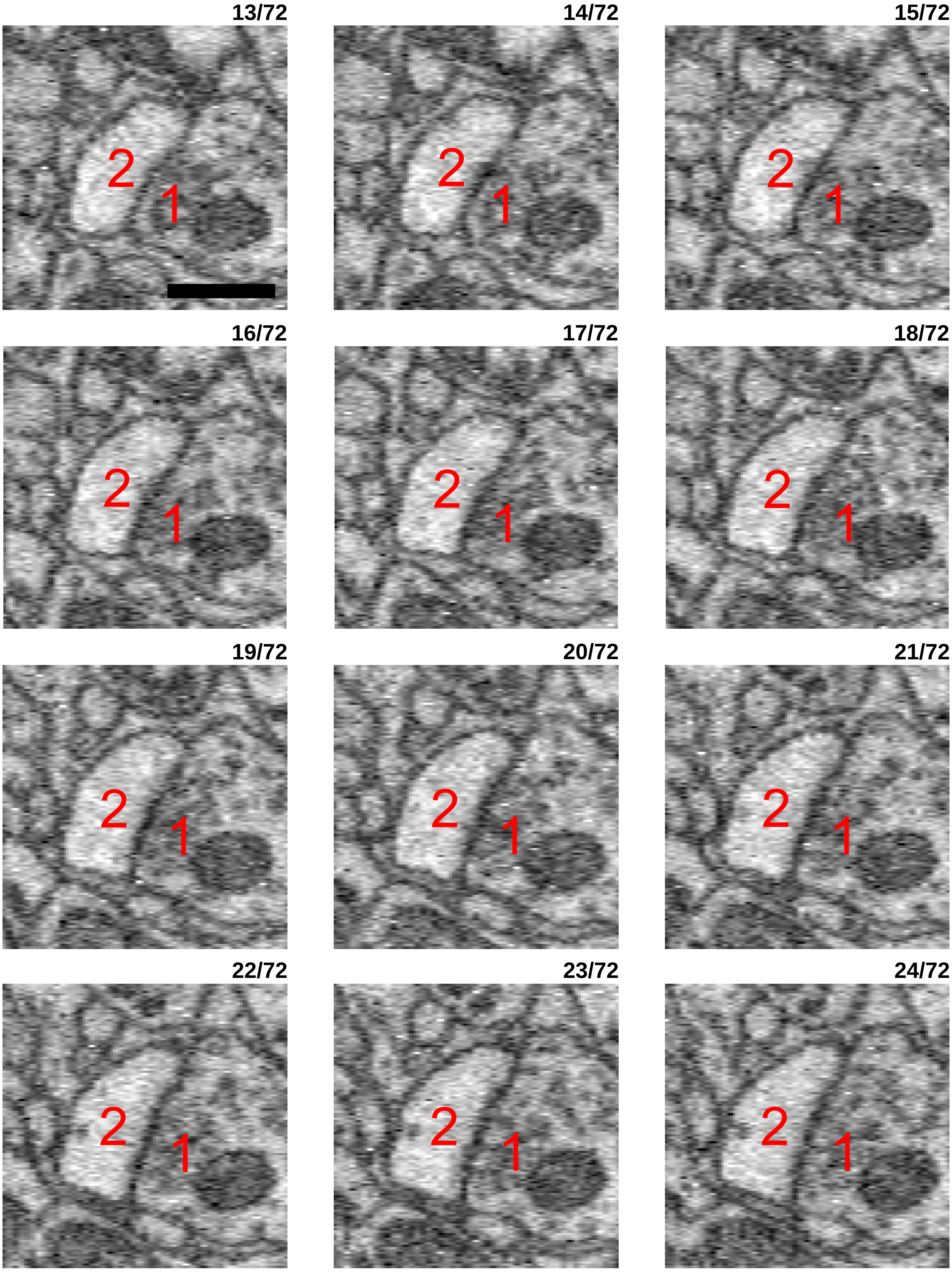

True positive (TP) synapse 03 - cont (3) yz orthogonal reslice

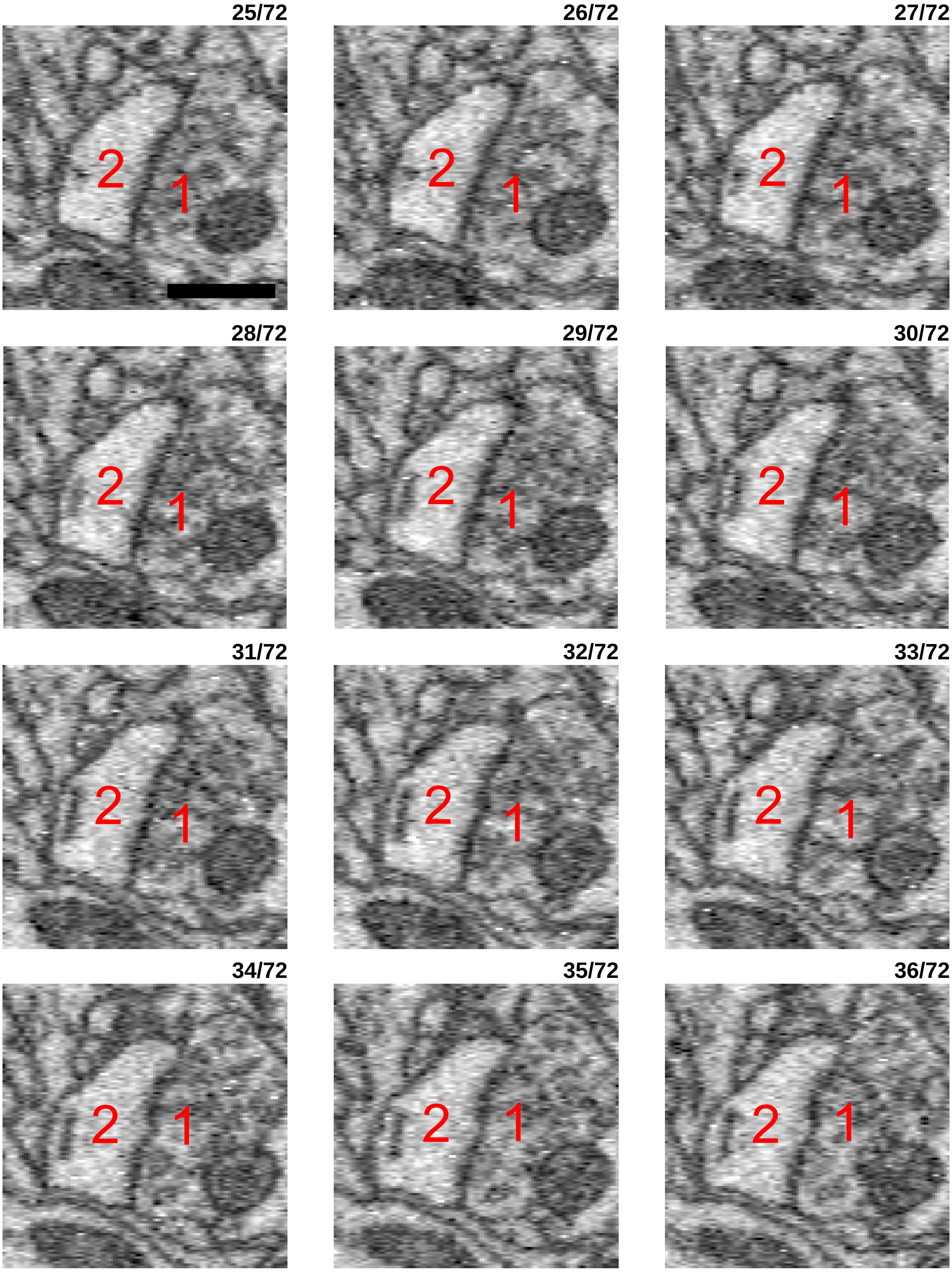

True positive (TP) synapse 03 - cont (4)

yz orthogonal reslice

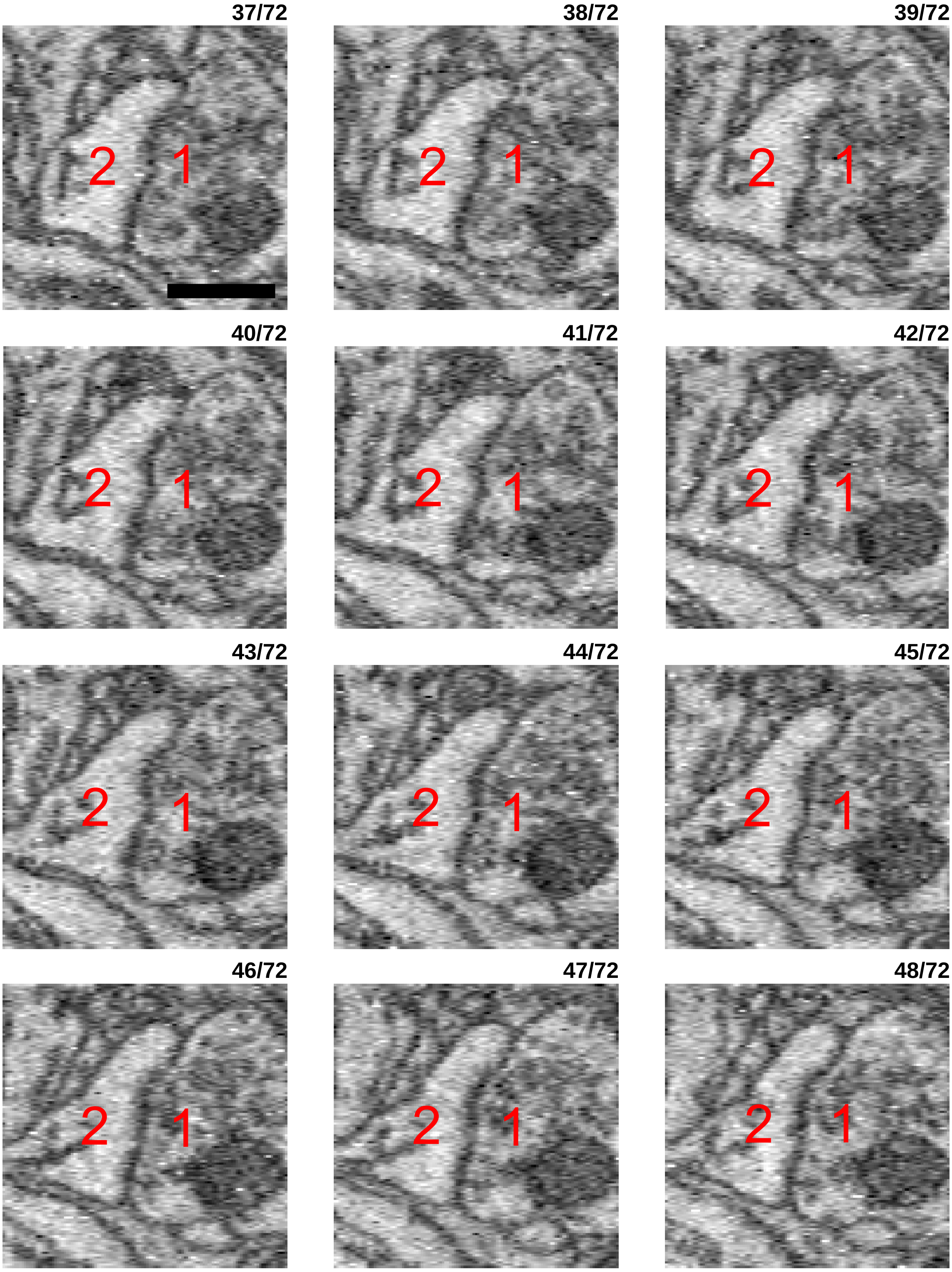

True positive (TP) synapse 03 - cont (5)

yz orthogonal reslice

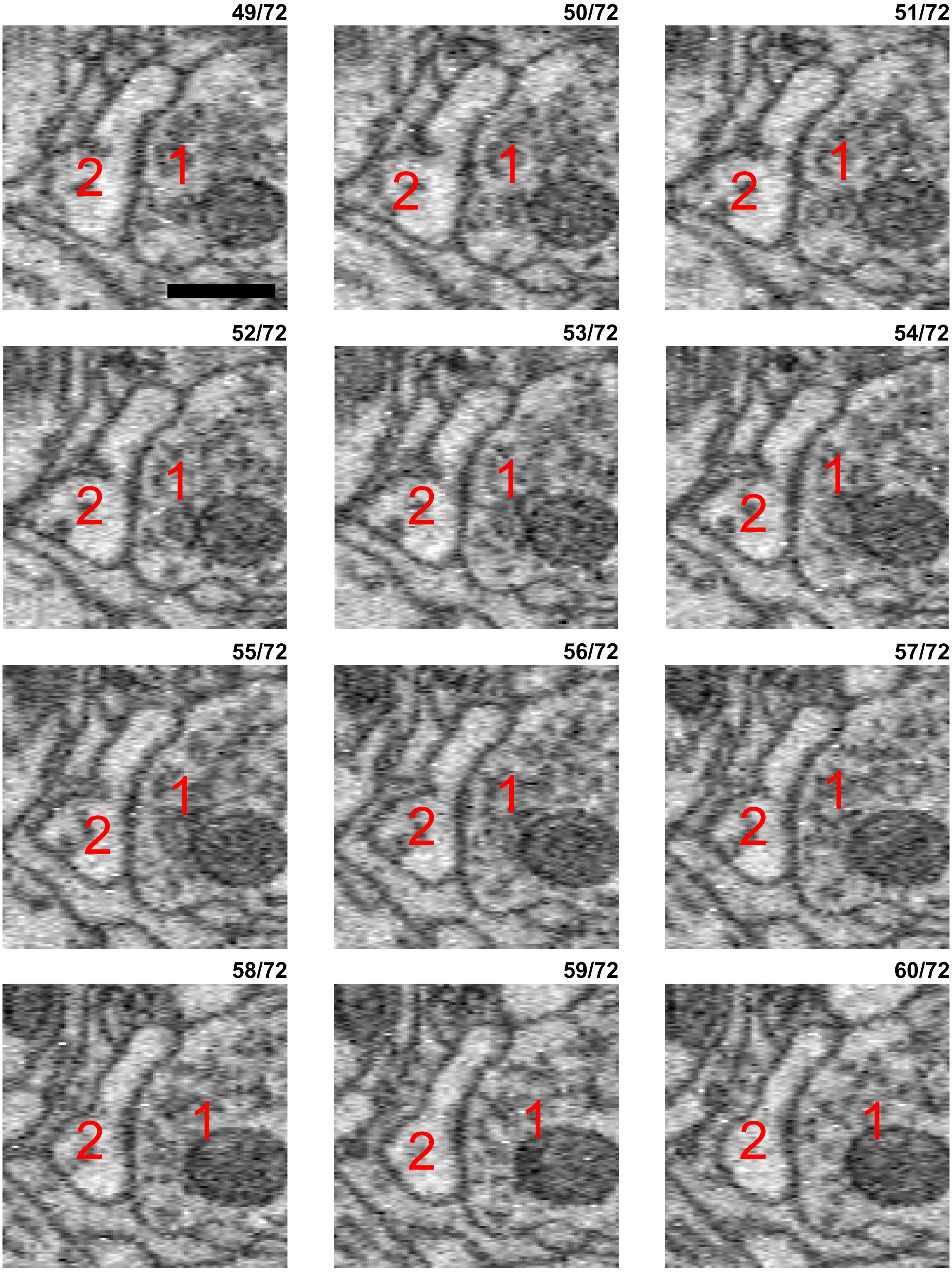

True positive (TP) synapse 03 - cont (6) yz orthogonal reslice

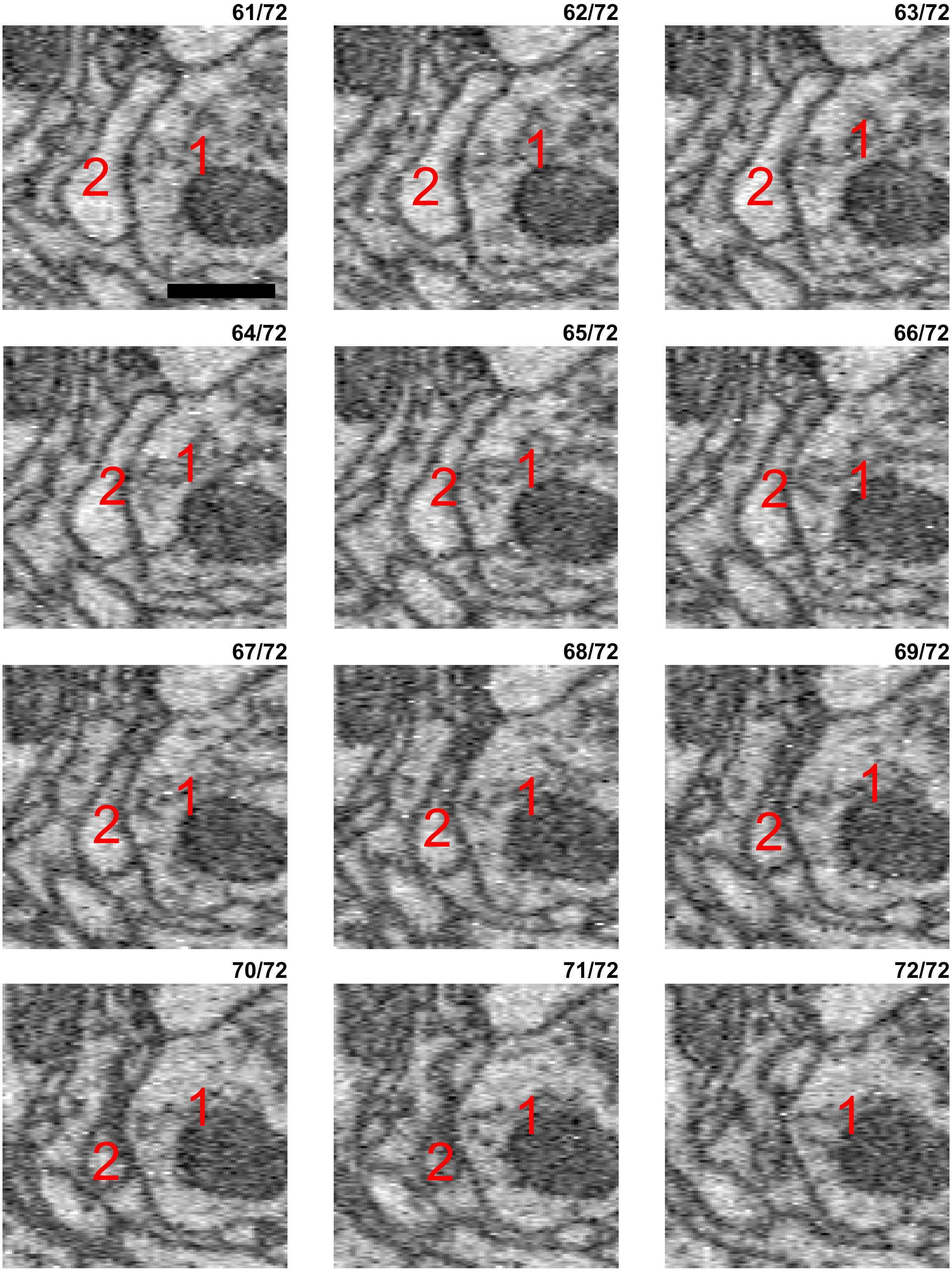

5 intermediate sized TP examples

(randomly selected)

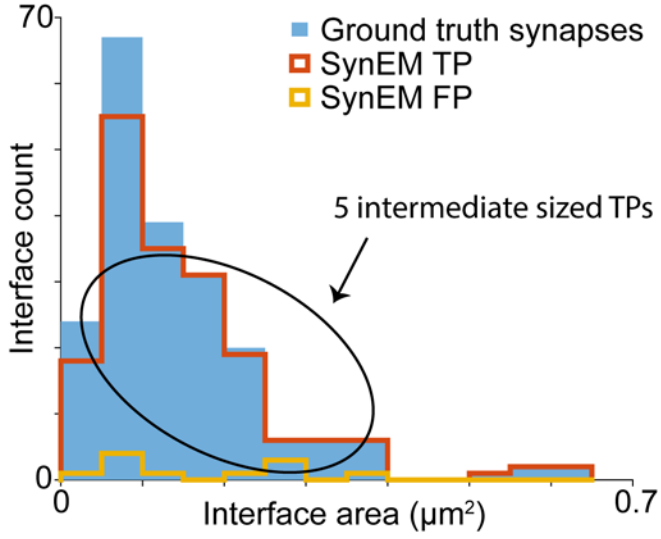

True positive (TP) synapse 04 xz orthogonal reslice

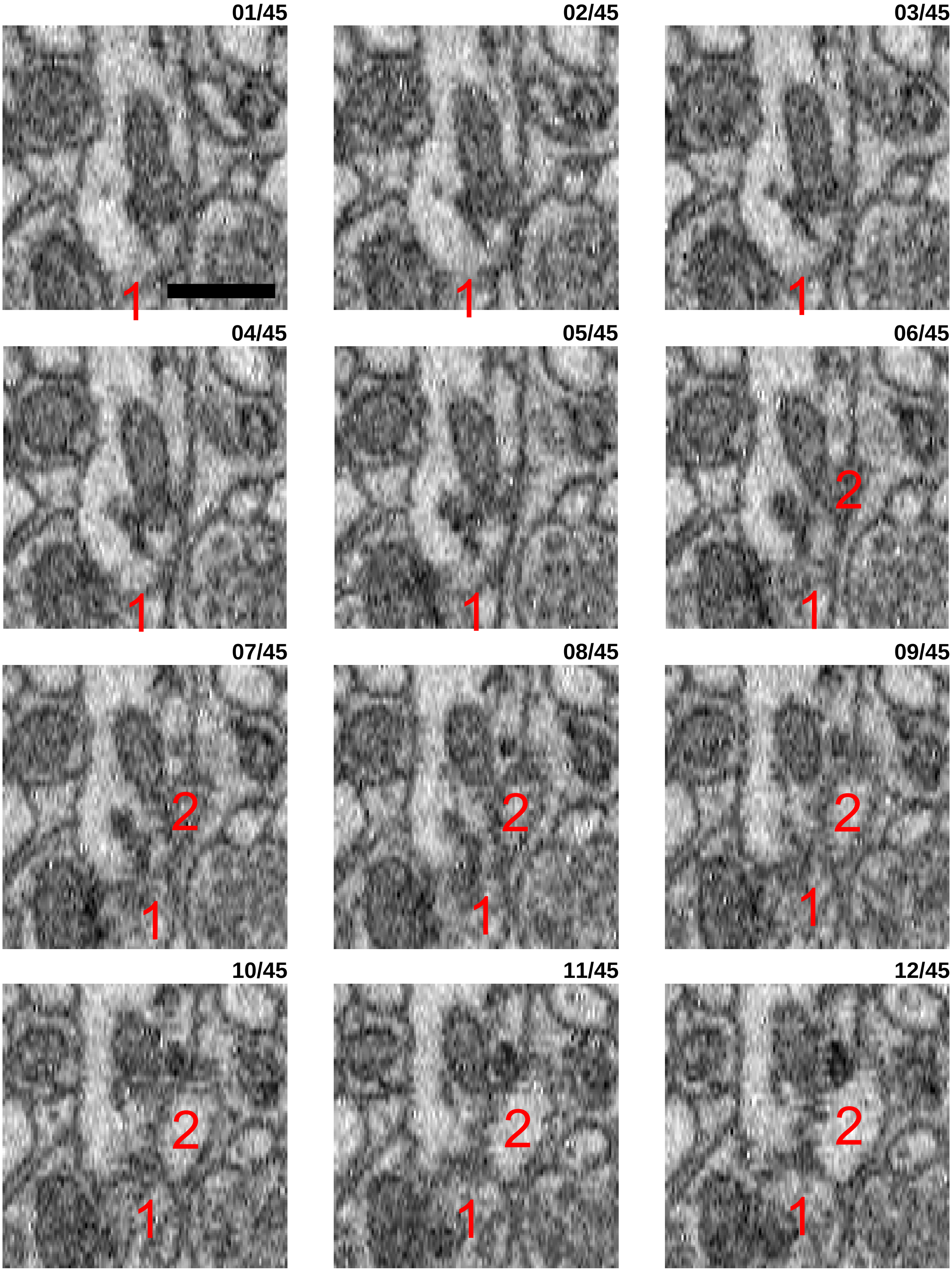

Medium sized synapse between a bouton (1, please see slices 30ff) and a spine head (2, also best visible in slices 30ff) shown in the xz orthogonal reslice. The vesicle cloud is starting to be visible at about slice 22 and is best visible around slices 27ff, where it approaches the synaptic membrane (slices 28-34). A darkening and slight broadening of the postsynaptic membrane can be also seen (most clearly in slice 33). Finally, the postsynaptic spine head has no obvious alternative innervation.

True positive (TP) synapse 04 - cont (2)

xz orthogonal reslice

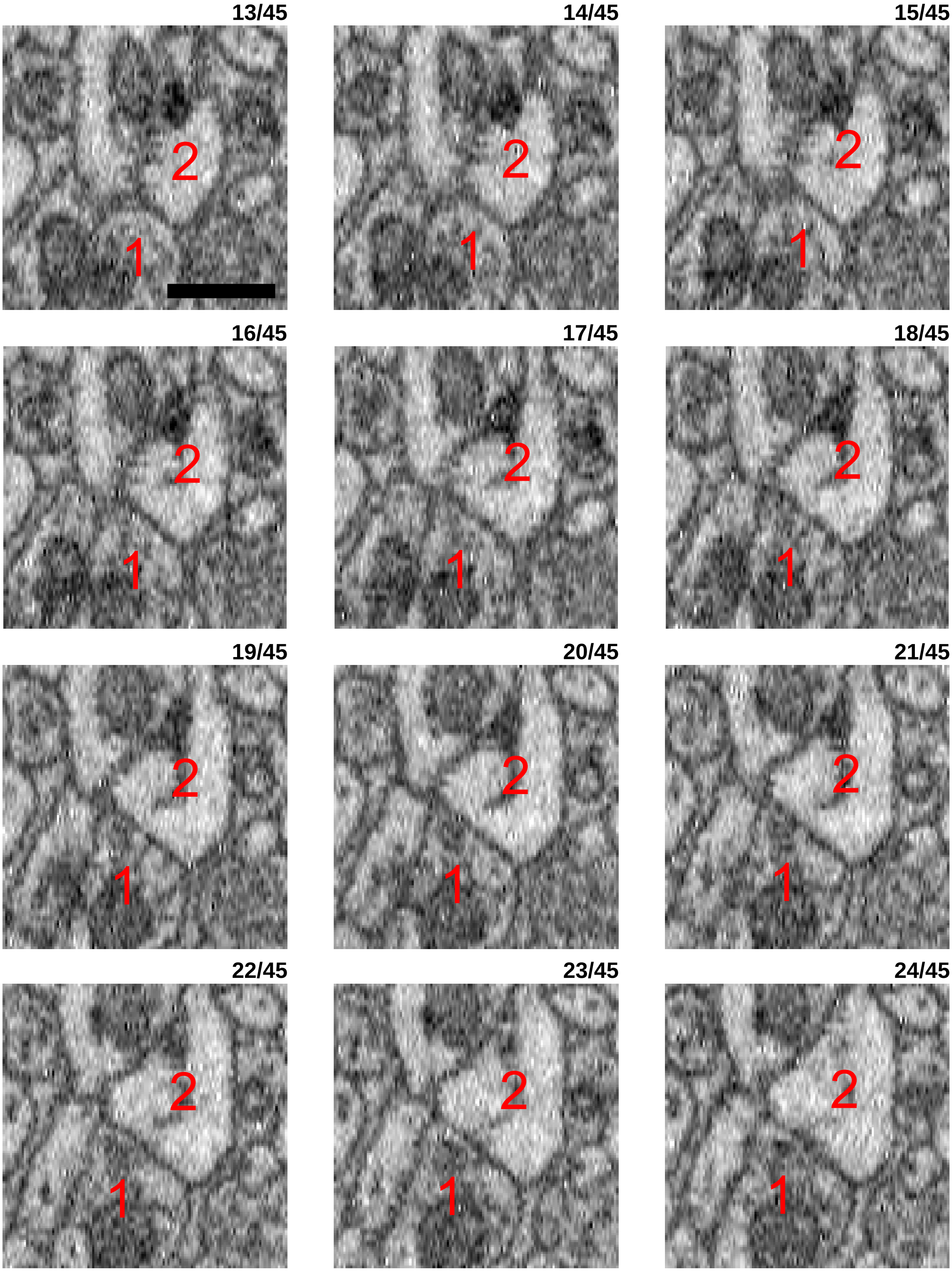

True positive (TP) synapse 04 - cont (3) xz orthogonal reslice

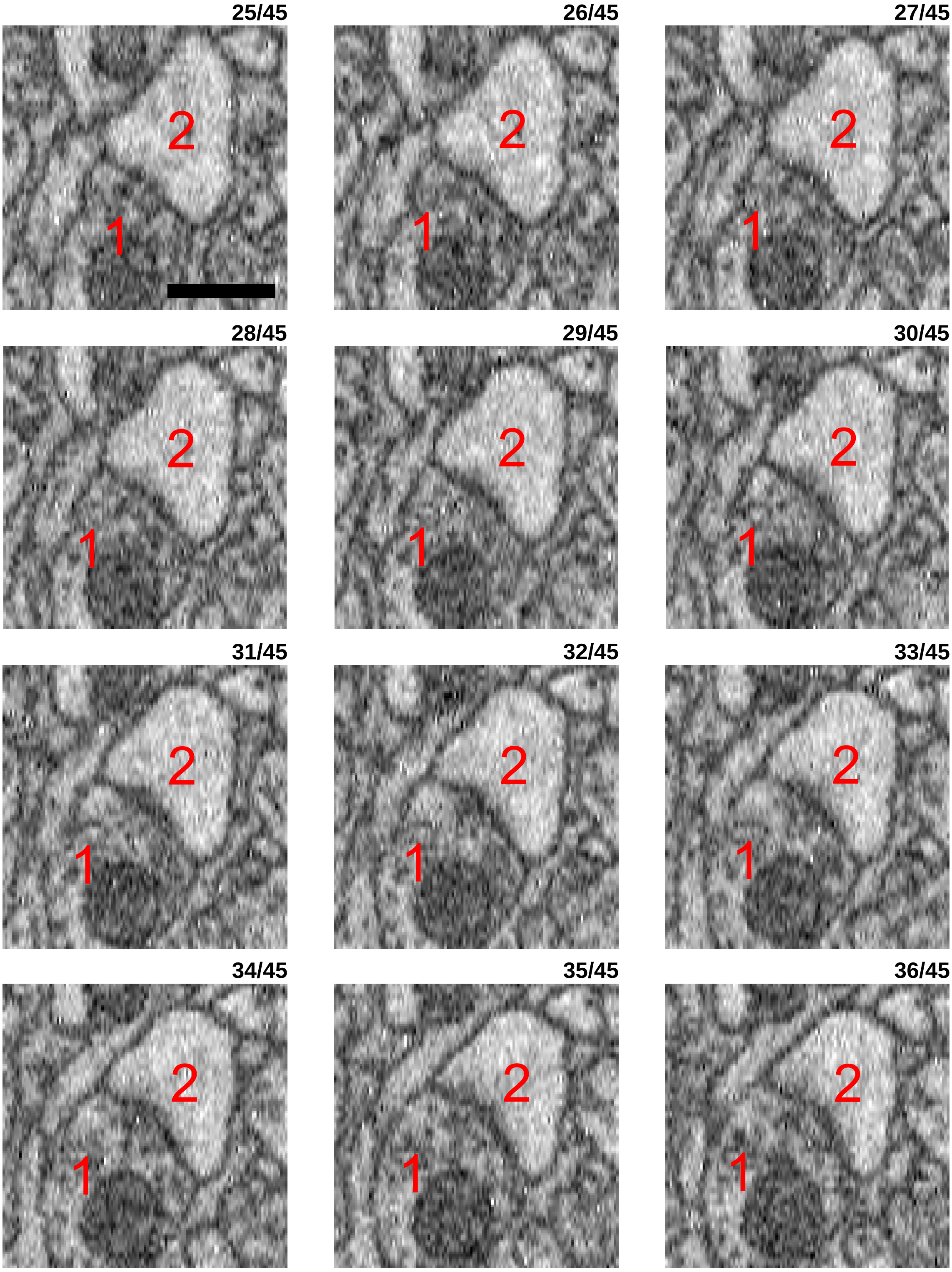

True positive (TP) synapse 04 - cont (4) xz orthogonal reslice

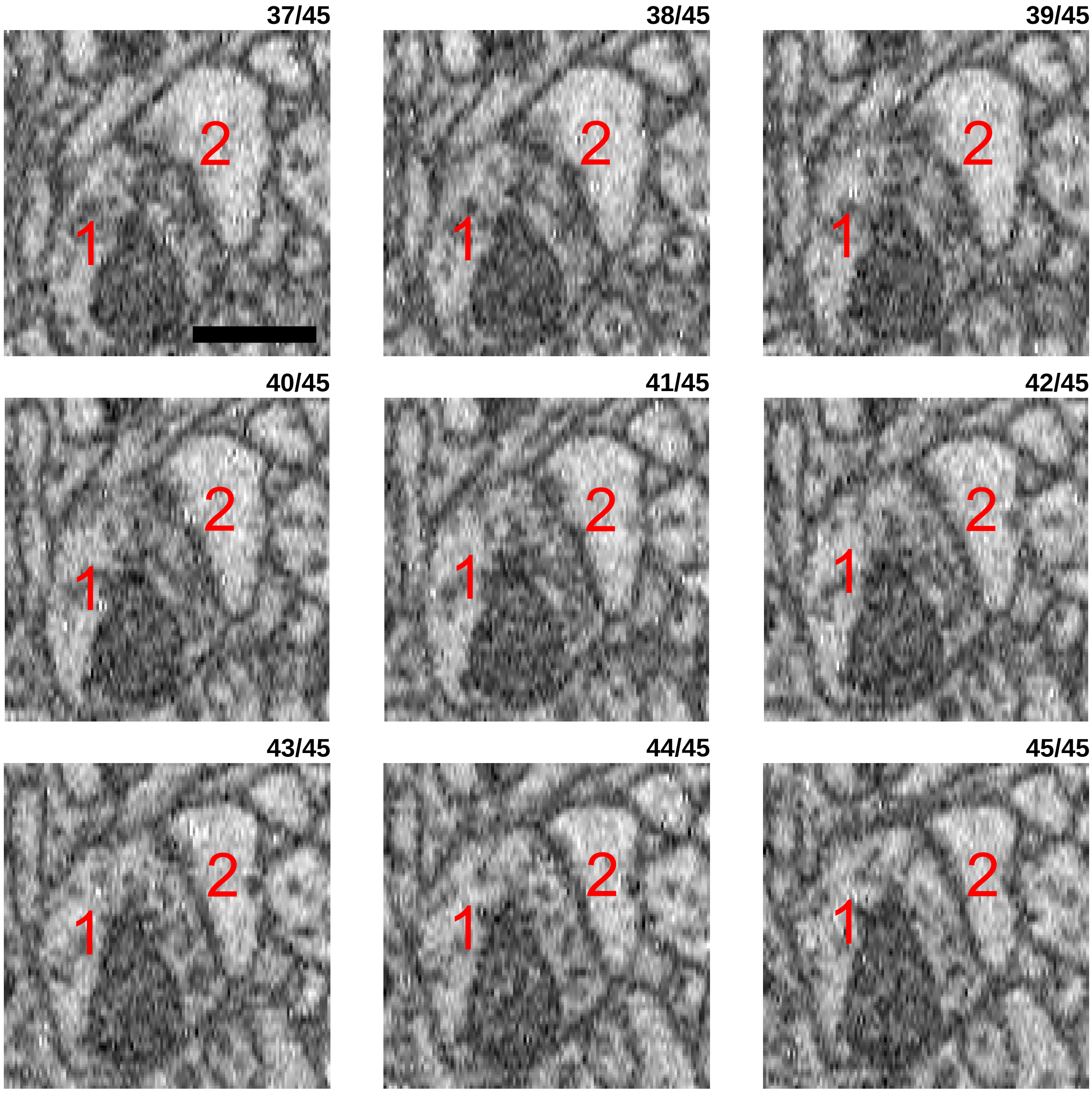

True positive (TP) synapse 05 xy imaging plane

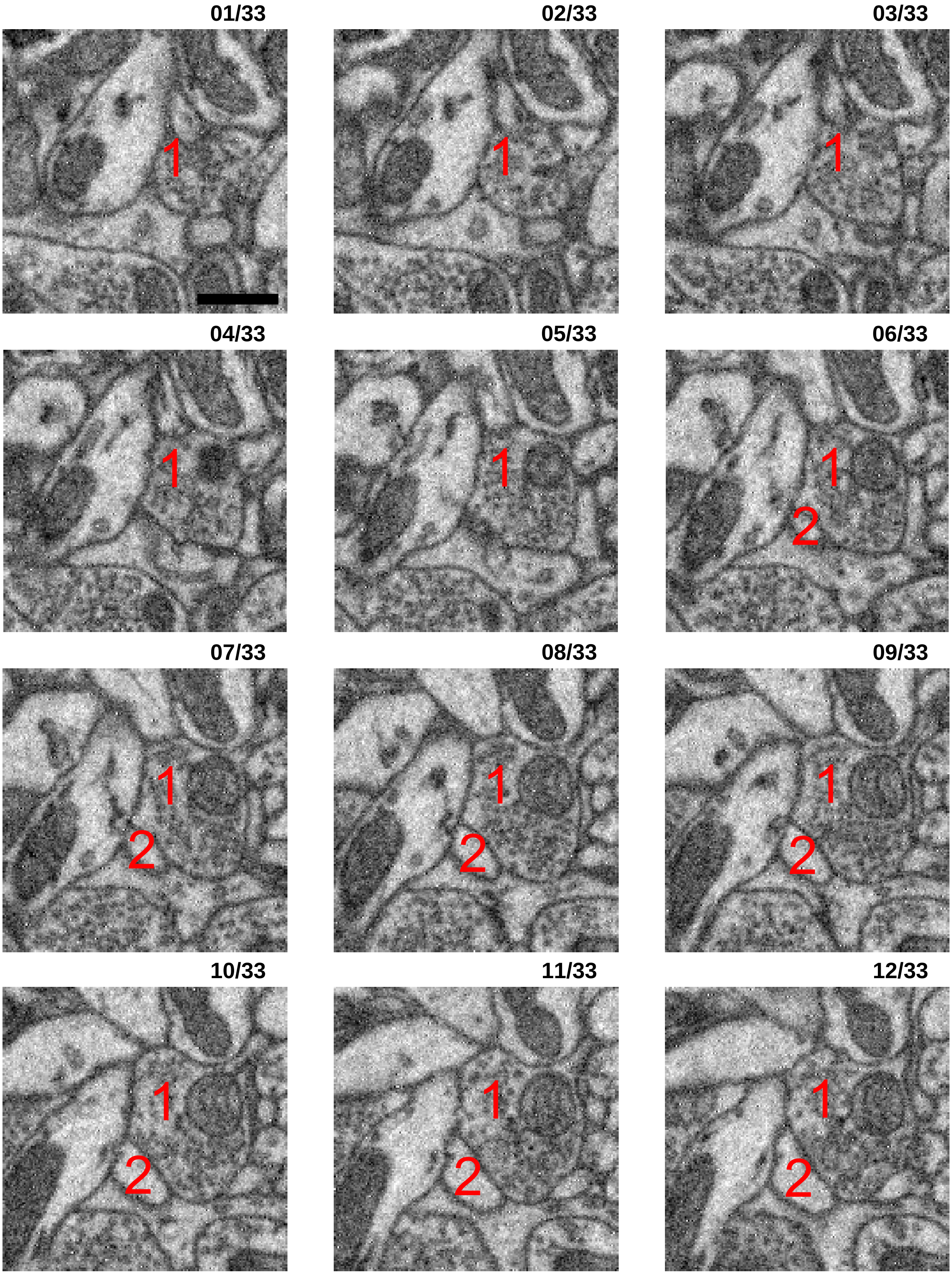

Large synapse between a large bouton (1) and a large spine head (2, slices 13 ff). The bouton is filled with many dozens of vesicles that approach the synaptic membrane (for example in slices 13ff), where the synaptic interface is also a bit darkened. Note that the apparent darkening and broadening of the synaptic membrane in the final slices 21ff is most likely due to the oblique cutting angle at that location. The postsynaptic spine head does not receive any alternative innervation (slices 6 to 33).

True positive (TP) synapse 05 - cont (2) xy imaging plane

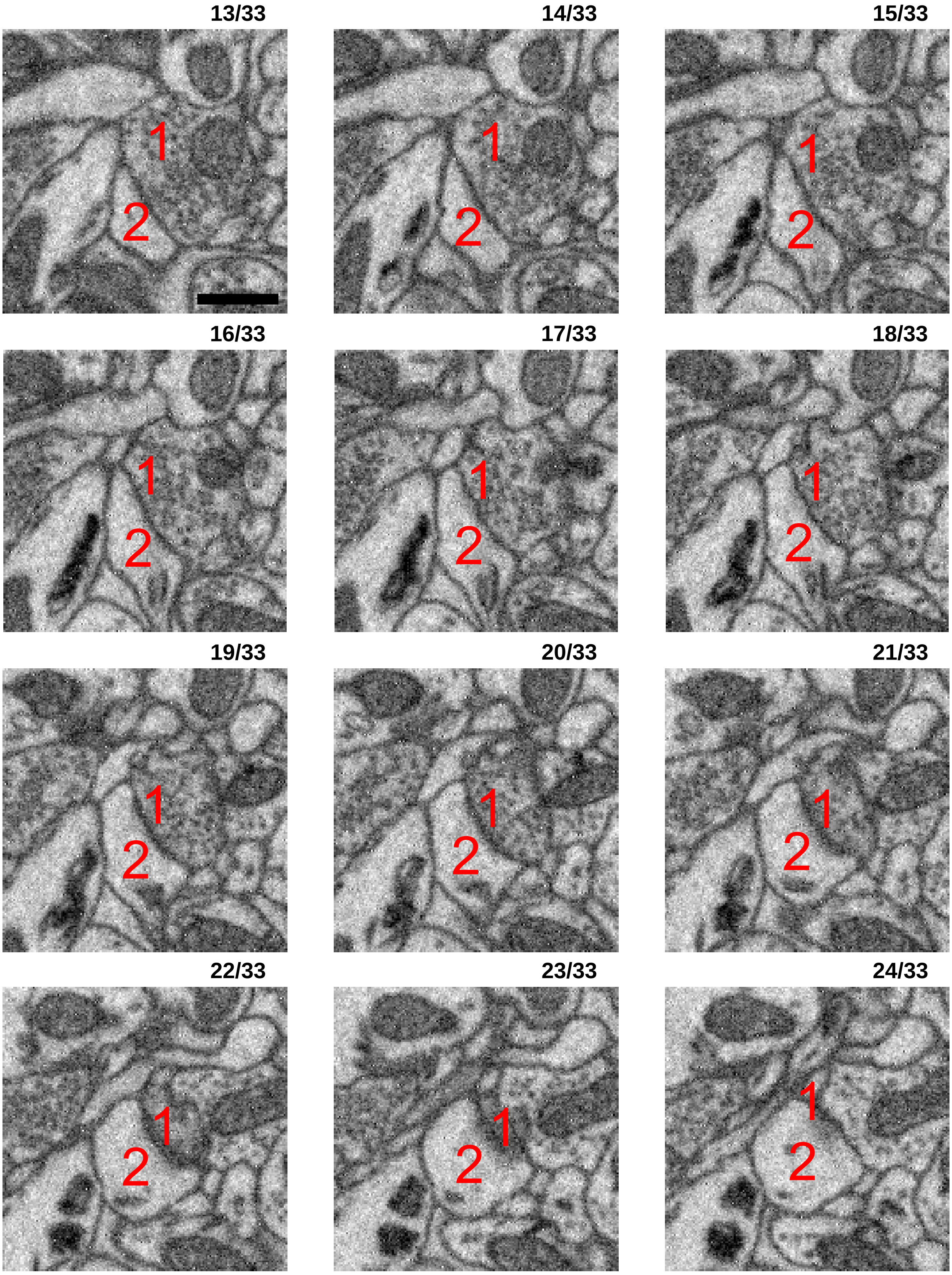

True positive (TP) synapse 05 - cont (3)

xy imaging plane

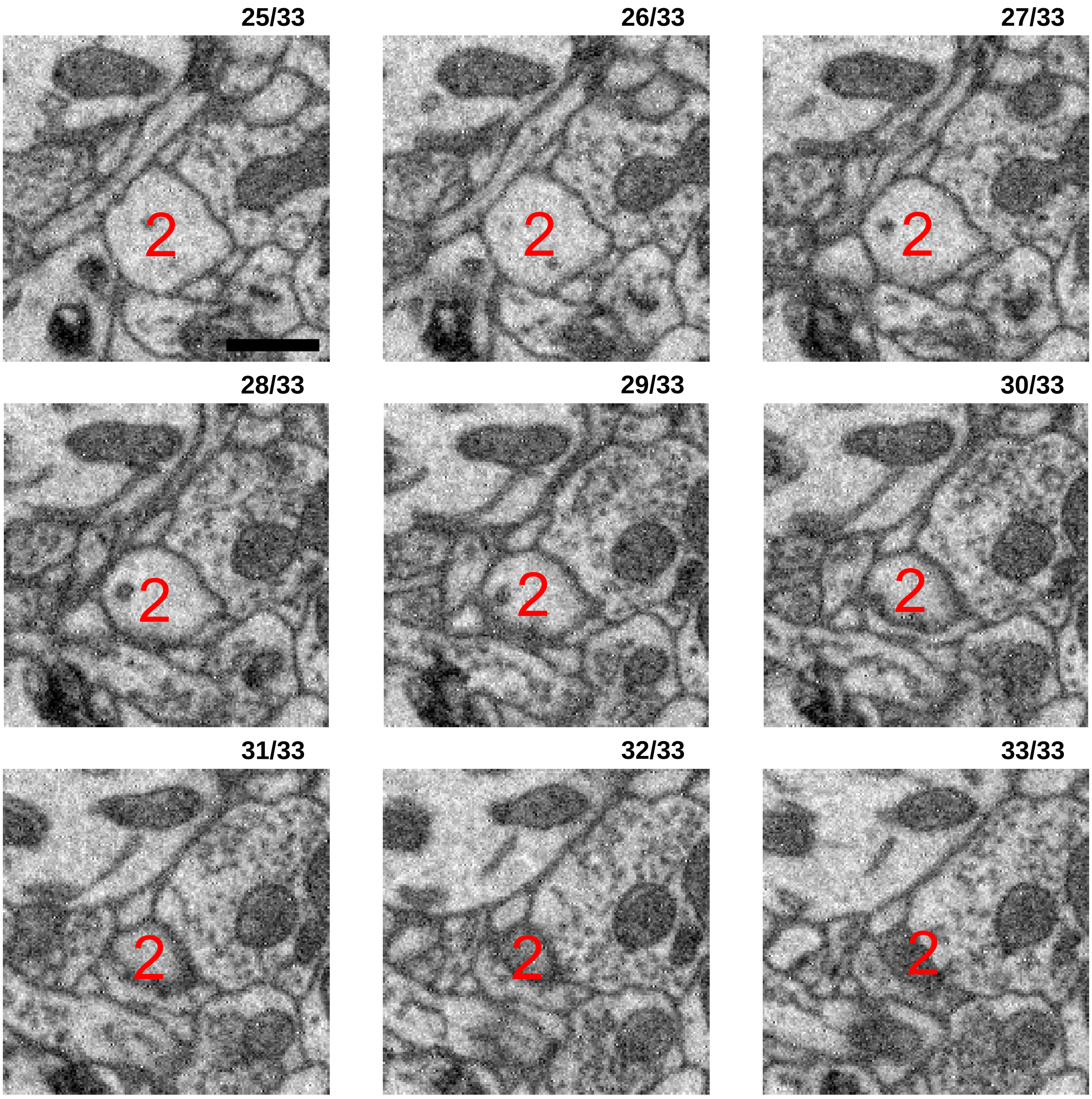

True positive (TP) synapse 06 xy imaging plane

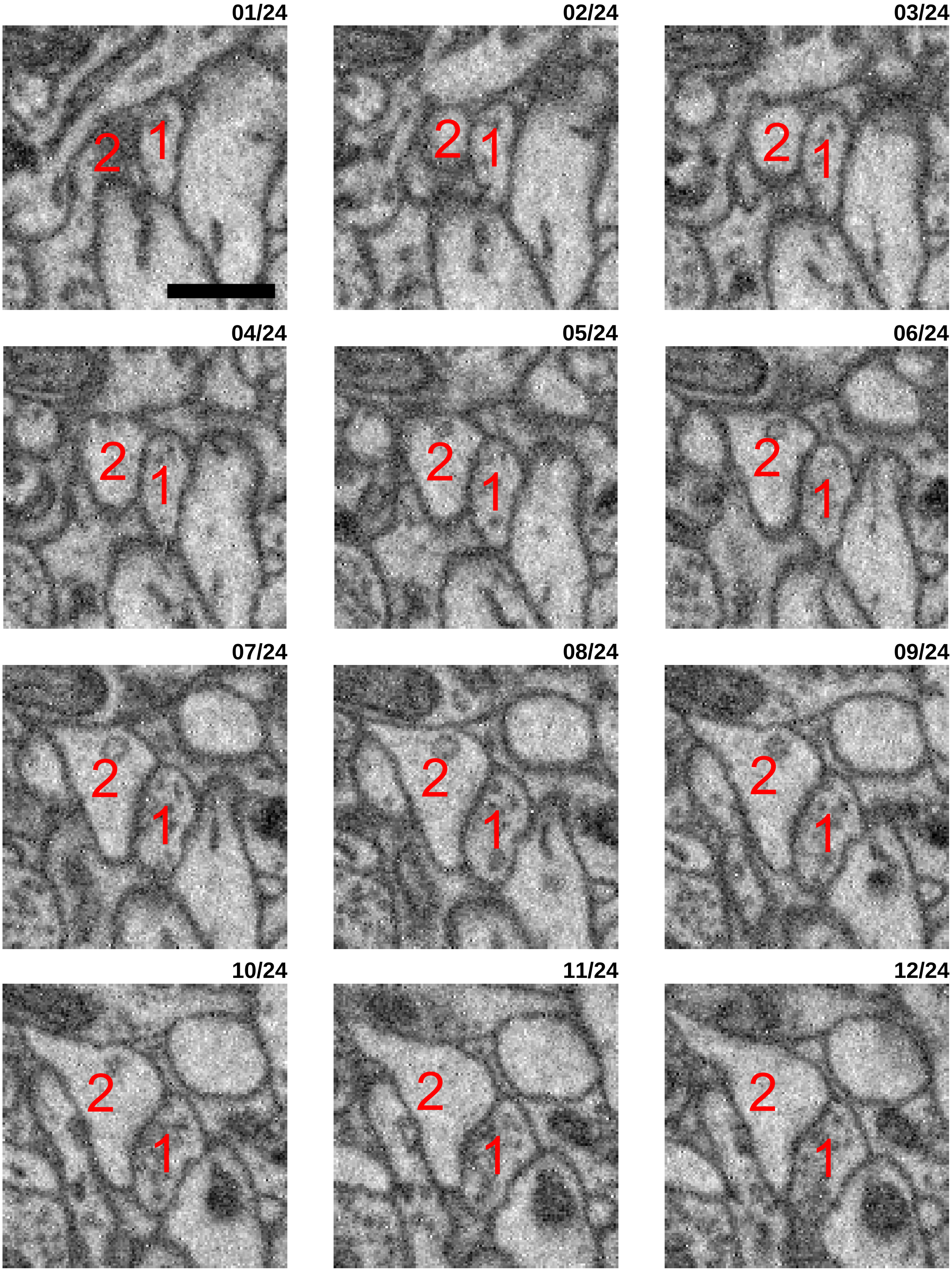

Small synapse between a presynaptic bouton (1), with maybe 2 dozen vesicles which approach the synaptic membrane (slices 10ff) together with a slight darkening and broadening of the membrane (slices 10 to 16), and a spine head (2). The postsynaptic spine head is not approached by any other competing axon (slices 1 to 24).

True positive (TP) synapse 06 - cont (2)   xy imaging plane

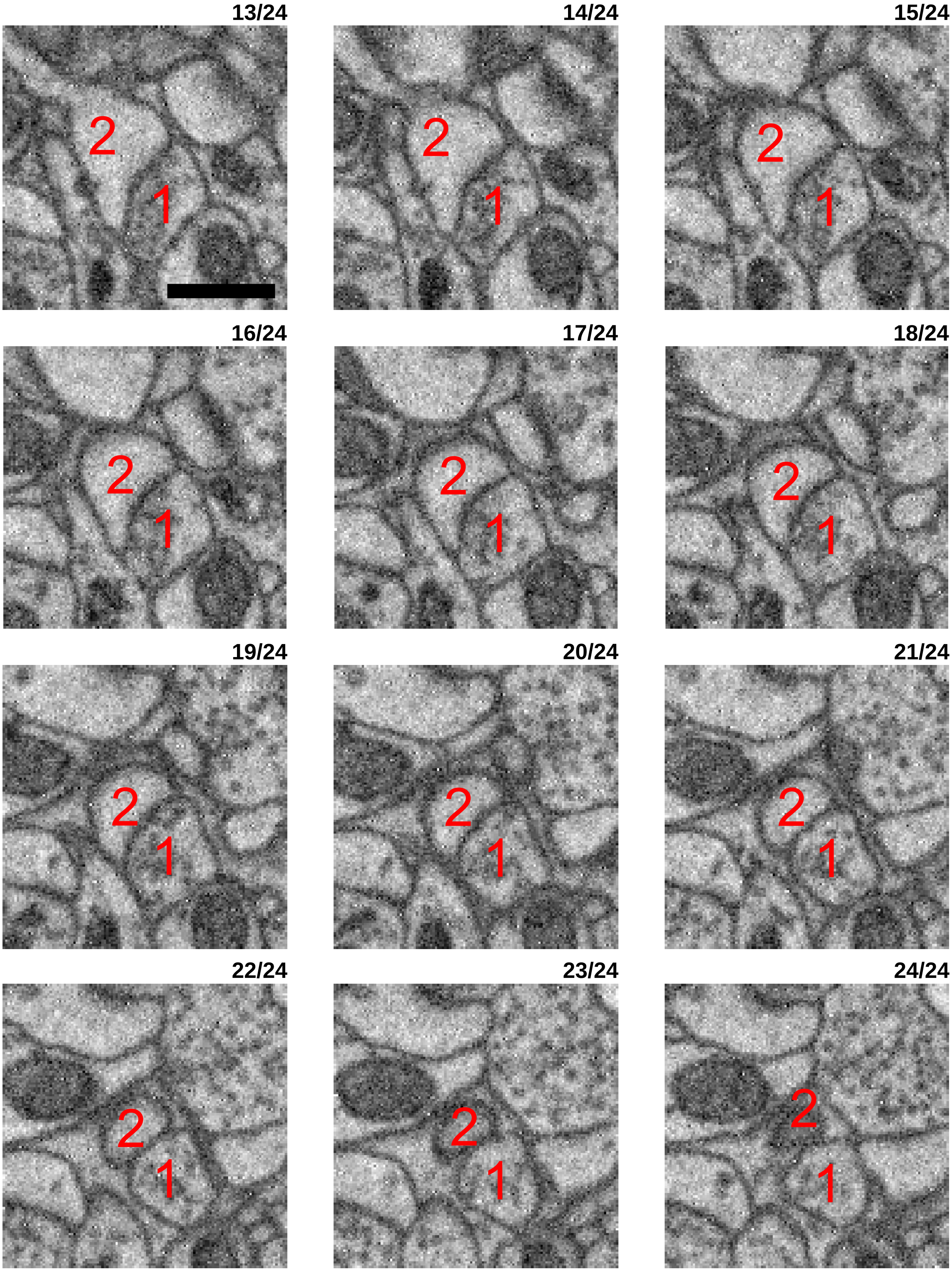

True positive (TP) synapse 07 xy imaging plane

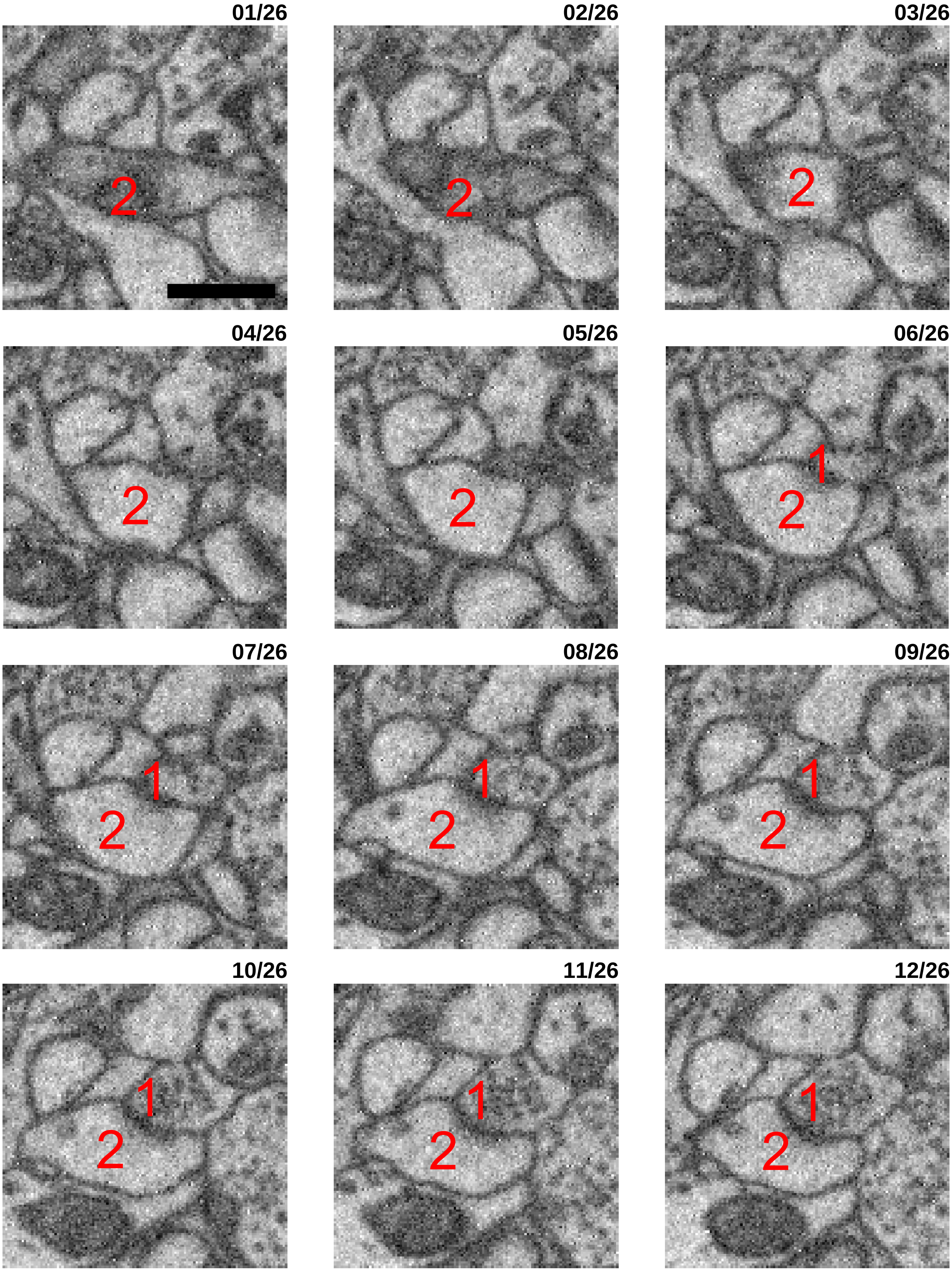

Synaptic contact between a small axonal bouton (1) and a spine head (2). The vesicle cloud approaches the membrane (visible in slices 11ff) together with a certain darkening and broadening of the membrane. Furthermore, the spine head does not receive a more plausible synaptic input. A second axonal bouton is approaching that same spine head (slices 9ff from the right). However, this bouton does neither show an attachment of vesicles to the membrane nor an accompanying darkening of the membrane.

True positive (TP) synapse 07 - cont (2)

xy imaging plane

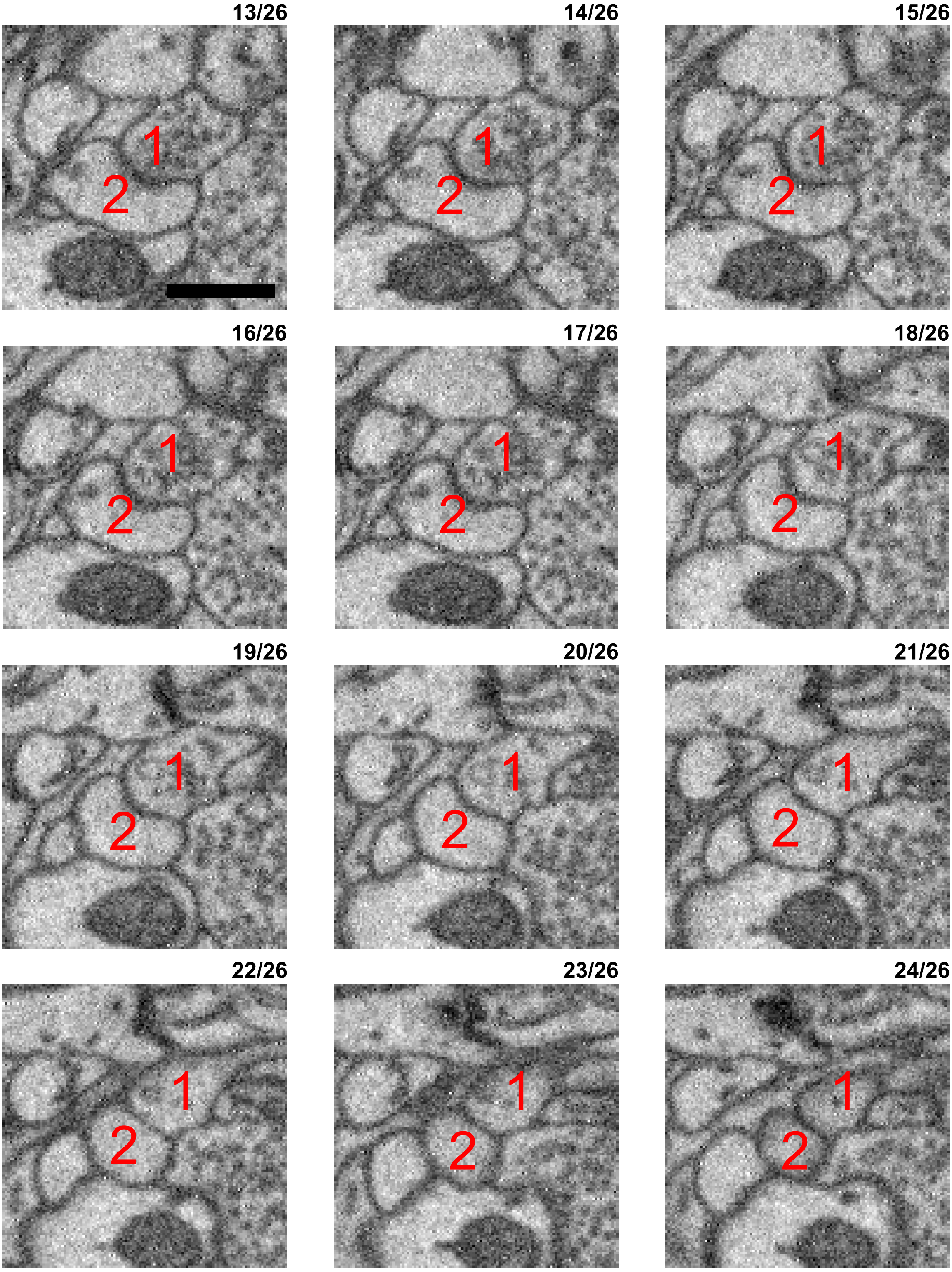

True positive (TP) synapse 07 - cont (3)

xy imaging plane

25/26

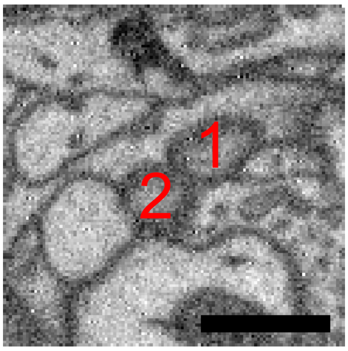

26/26

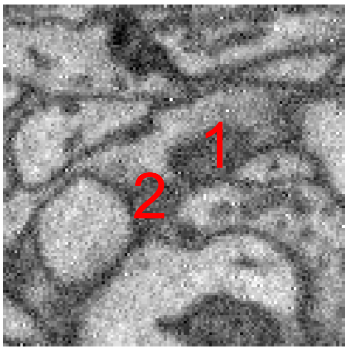

True positive (TP) synapse 08 xy imaging plane

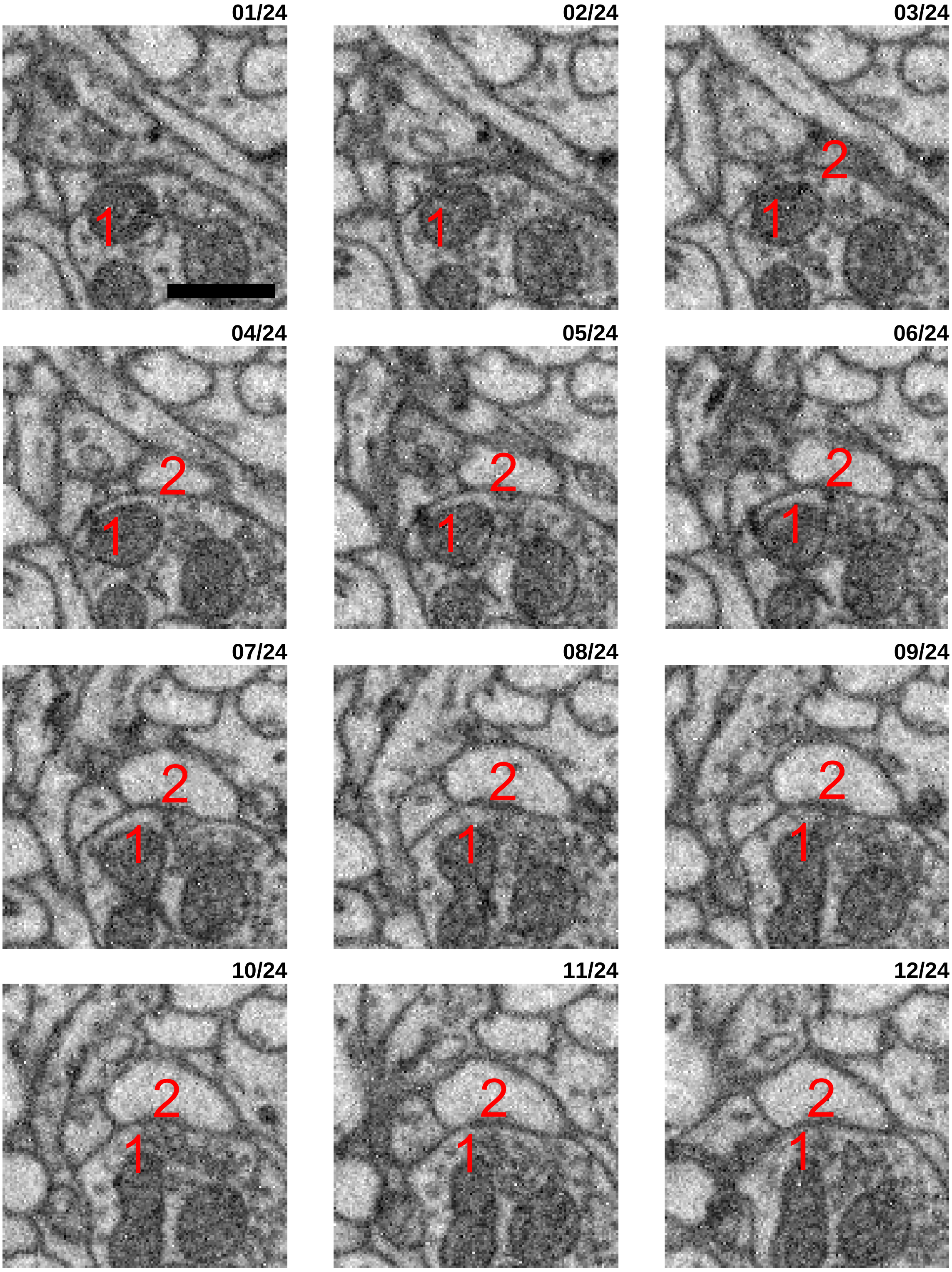

Synaptic contact between a large bouton (1) and a small spine head (2). The bouton is filled with two large mitochondria (visible in all slices) and dozens of vesicles. These vesicles approach the membrane of interest (visible in slices 7ff) together with a slight broadening and darkening of this membrane. The spine head is not approached by a competing axonal input. Note, however, that the axonal bouton (1) makes a second synapse visible in slices 18ff to a second spine head (S) just below the synapse from (1) to (2). The synapse from (1) to (S) is also a TP detection.

True positive (TP) synapse 08 - cont (2) xy imaging plane

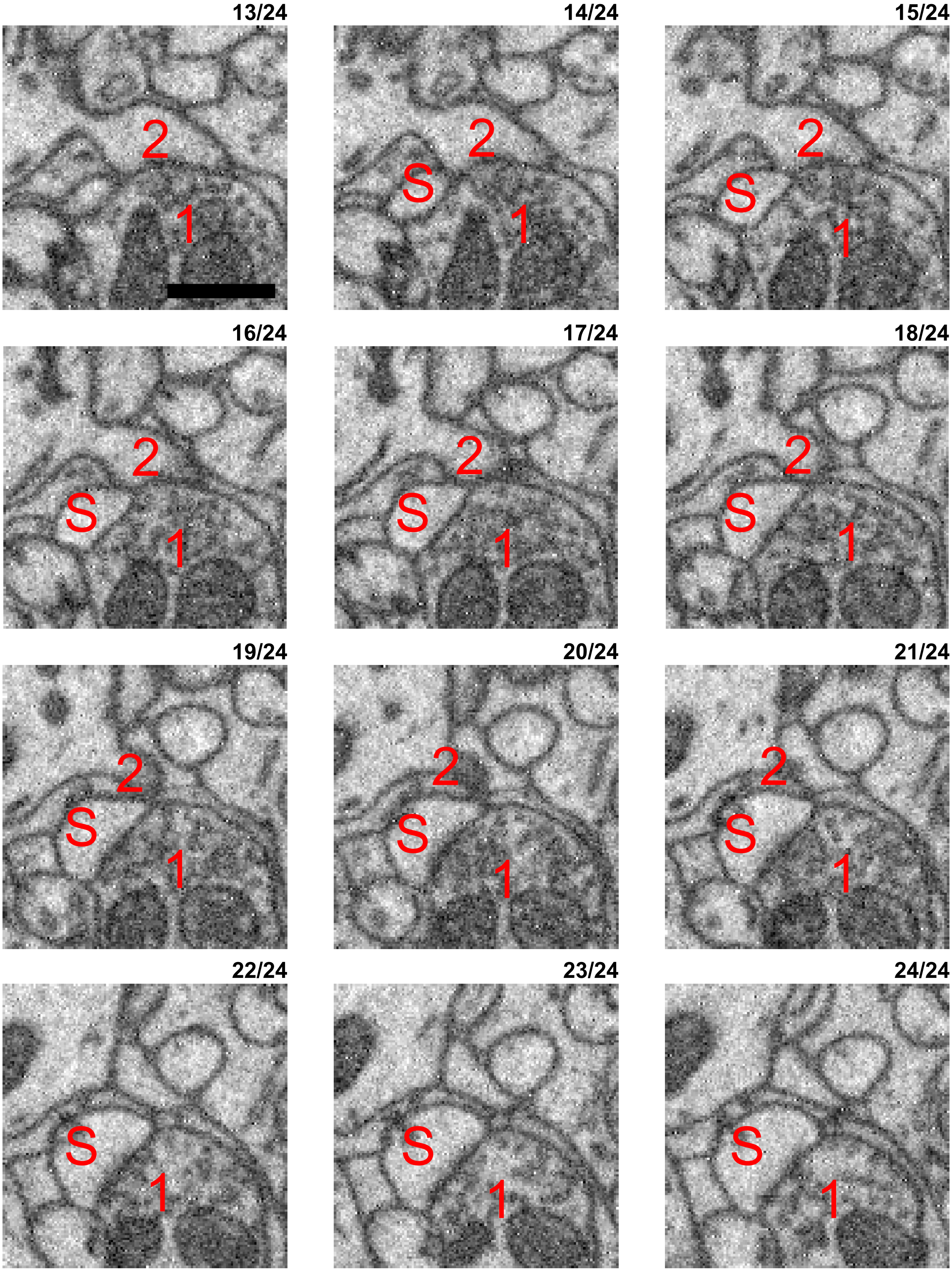

3 smallest TP examples

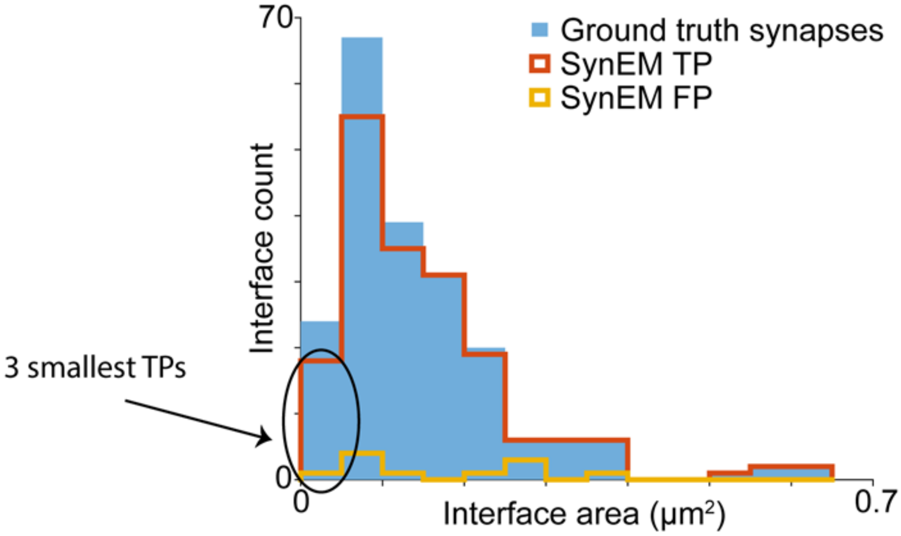

Note that the following interfaces are the 3 smallest interfaces that have been labeled as a synapse by both human experts and the SynEM classifier. As reported above (page 8), both human and SynEM detection performance becomes substantially lower for these very small interfaces (see the 2nd and 3rd of the following examples for illustration).

True positive (TP) synapse 09 xy imaging plane

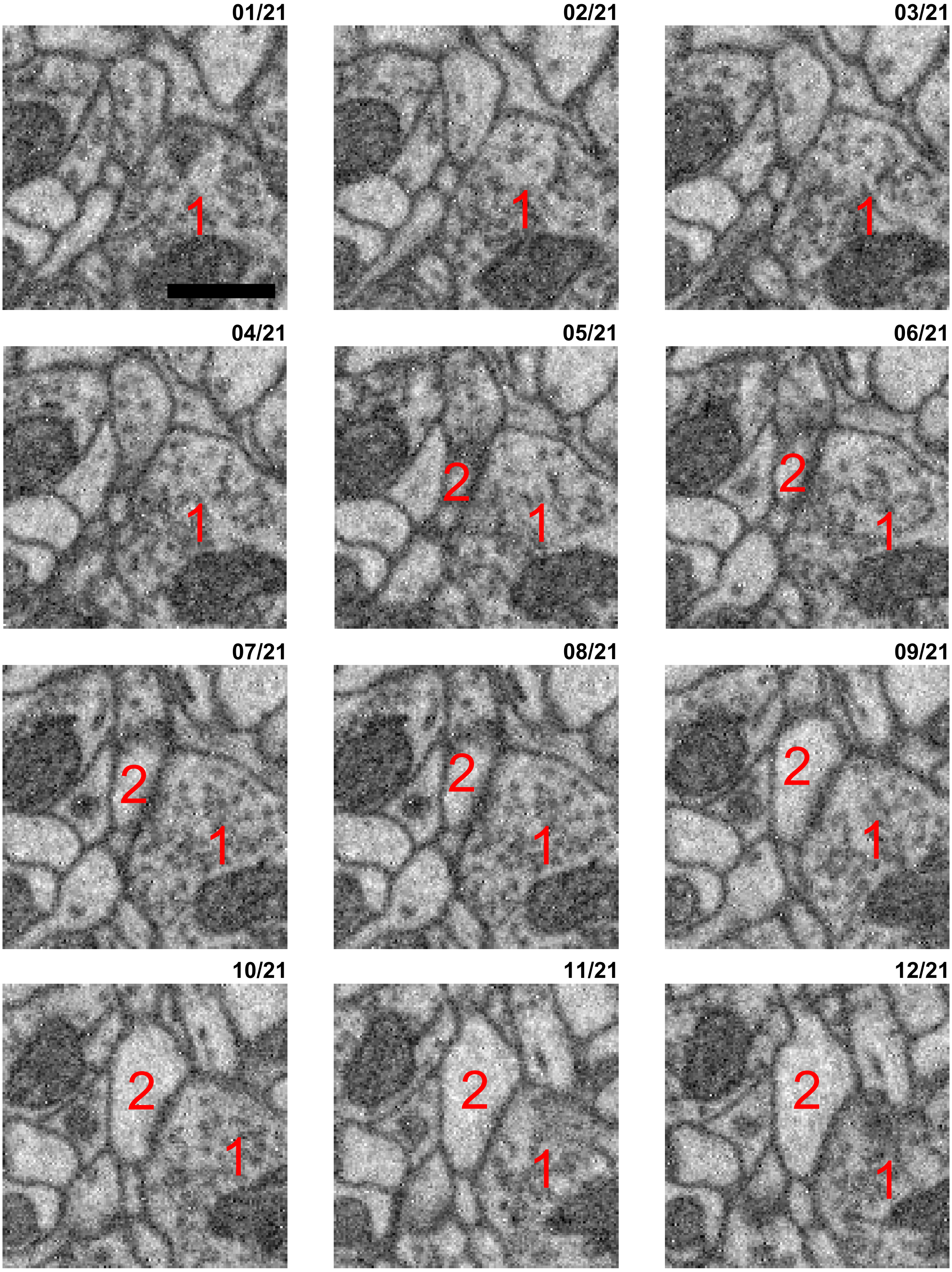

Medium size synaptic contact between a presynaptic bouton (1) and a medium sized spine head (2). The bouton is filled with a few dozen vesicles which come close to the membrane (slices 6, 7 and 8) together with a certain widening and darkening of the interface (especially in slices 9 and 10). The spine head does not receive input from a competing axon (slices 1 to 21).

True positive (TP) synapse 09 - cont (2) xy imaging plane

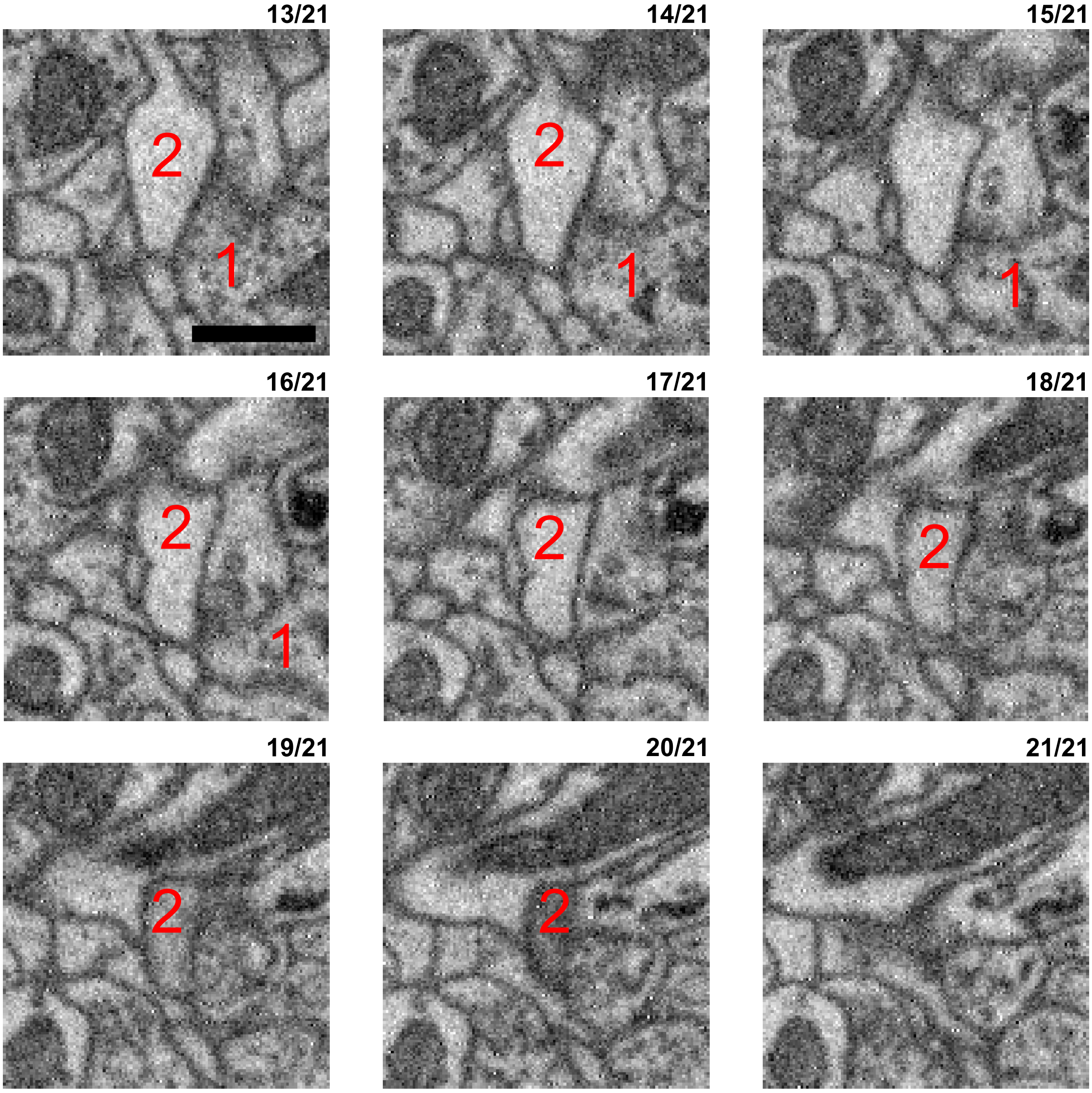

True positive (TP) synapse 10 yz orthogonal reslice

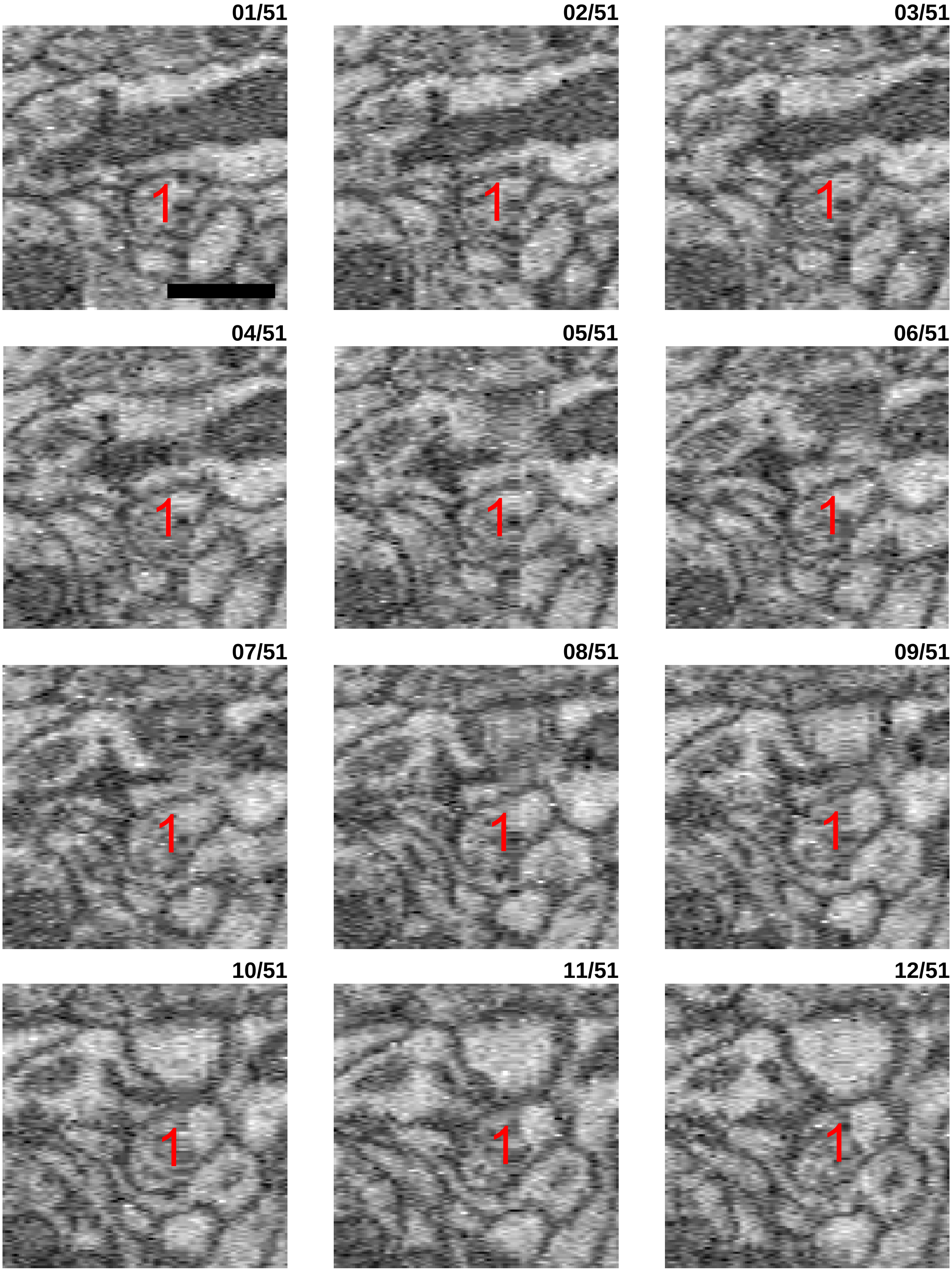

Very small contact between an axon (1) and a small spine head (2) shown in the yz orthogonal reslice. The vesicle cloud and the bouton are only hardly distinguishable and must be inferred from the fact that this is an axonal process (slices 10ff). The spine head (2) can be detected in slices 10 to about 40. If the darker gray cloud in slices 13ff within (1) is a vesicle cloud then the most likely partner is the spine head (2). The only other process in proximity to this vesicle cloud is the process below (1) (visible in slices 15ff), which is however a glia cell. The spine head (2) is

True positive (TP) synapse 10 - cont (2) yz orthogonal reslice

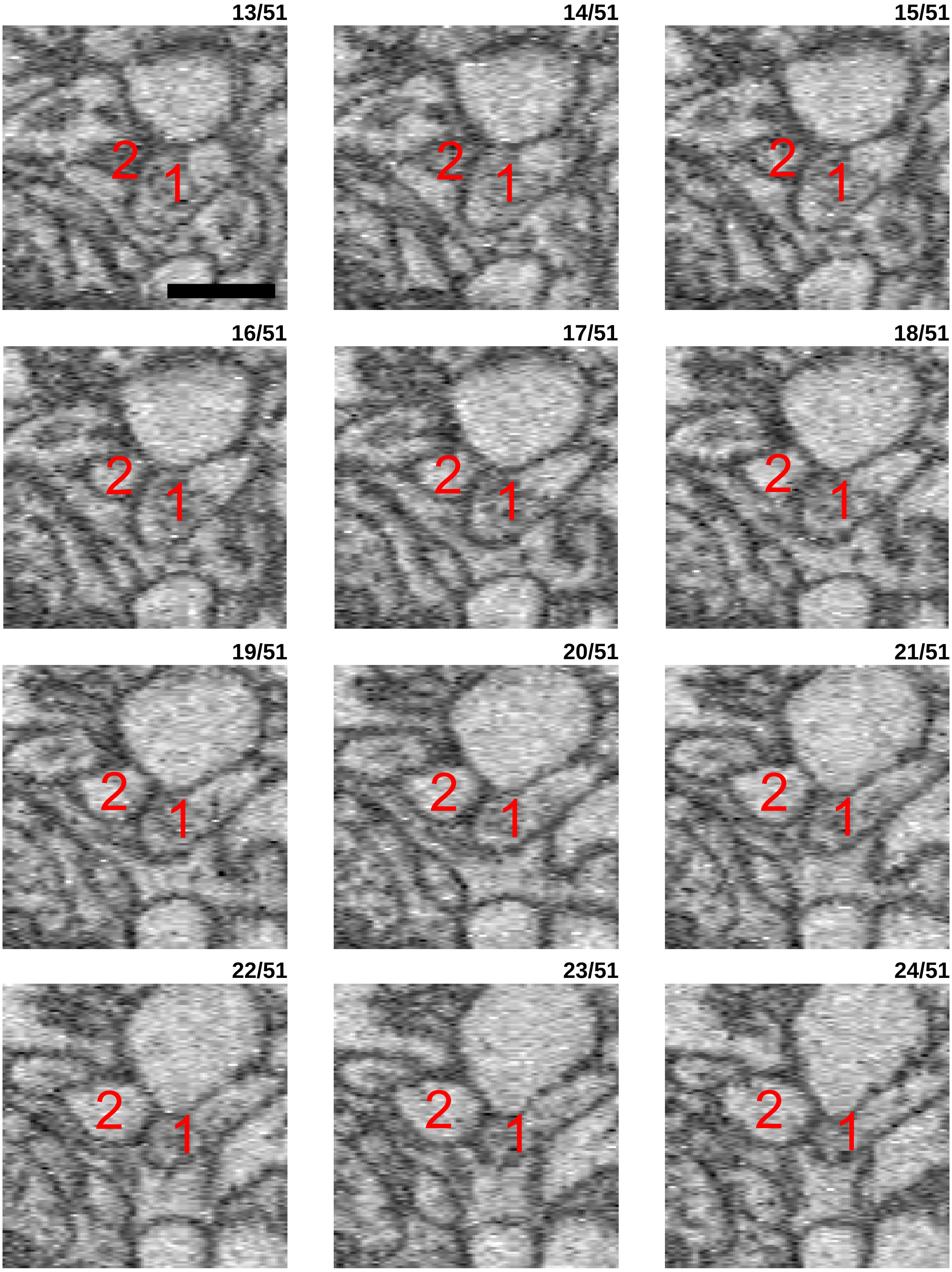

not approached by any other candidate axon (slices 13-51). Based on these considerations the most likely conclusion is that bouton (1) innervates spine head (2). Please note that, as discussed in the introduction to this document, this is the second smallest synapse that was agreed upon by human experts, and this is in a size range where synapse detection agreement is substantially reduced for experts.

True positive (TP) synapse 10 - cont (3)

yz orthogonal reslice

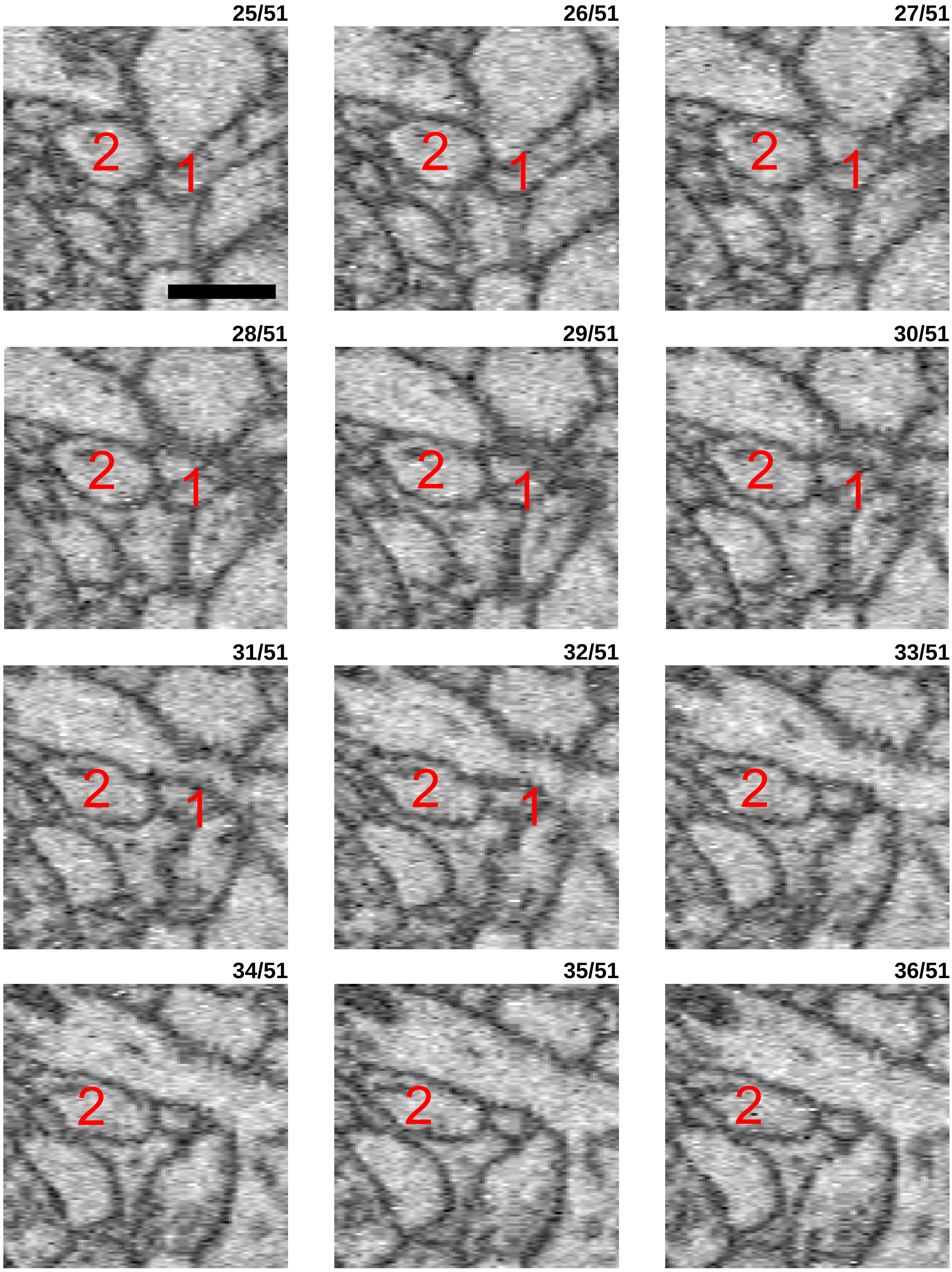

True positive (TP) synapse 10 - cont (4)

yz orthogonal reslice

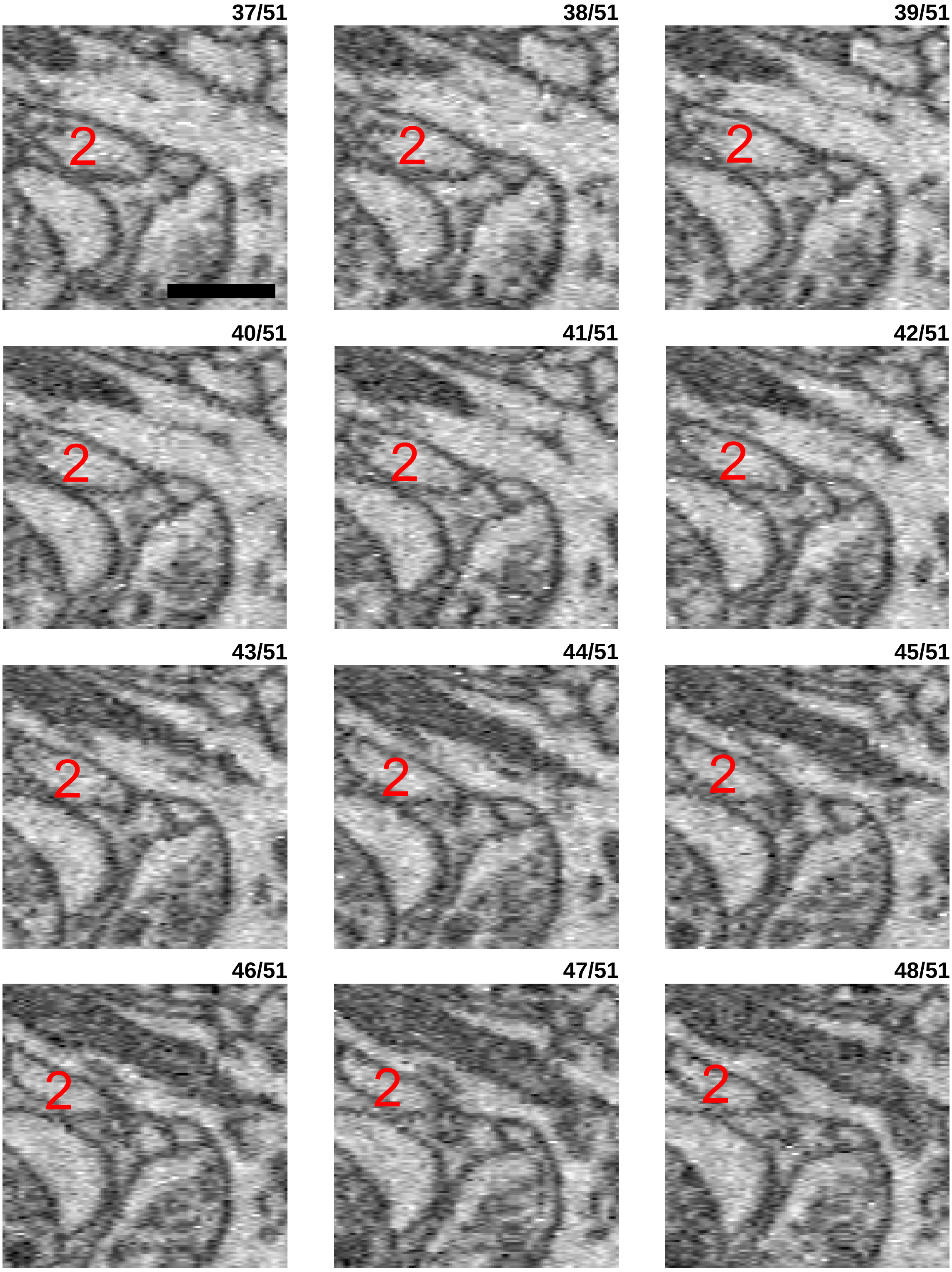

True positive (TP) synapse 10 - cont (5) yz orthogonal reslice

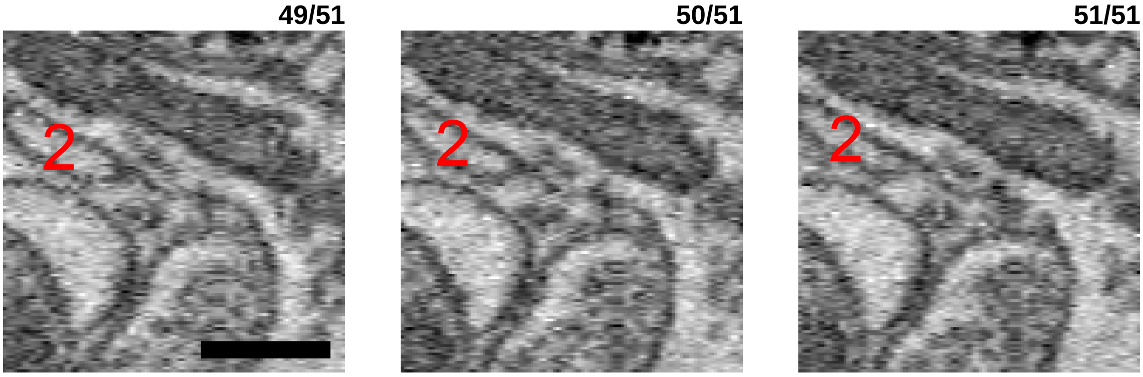

True positive (TP) synapse 11 xz orthogonal reslice

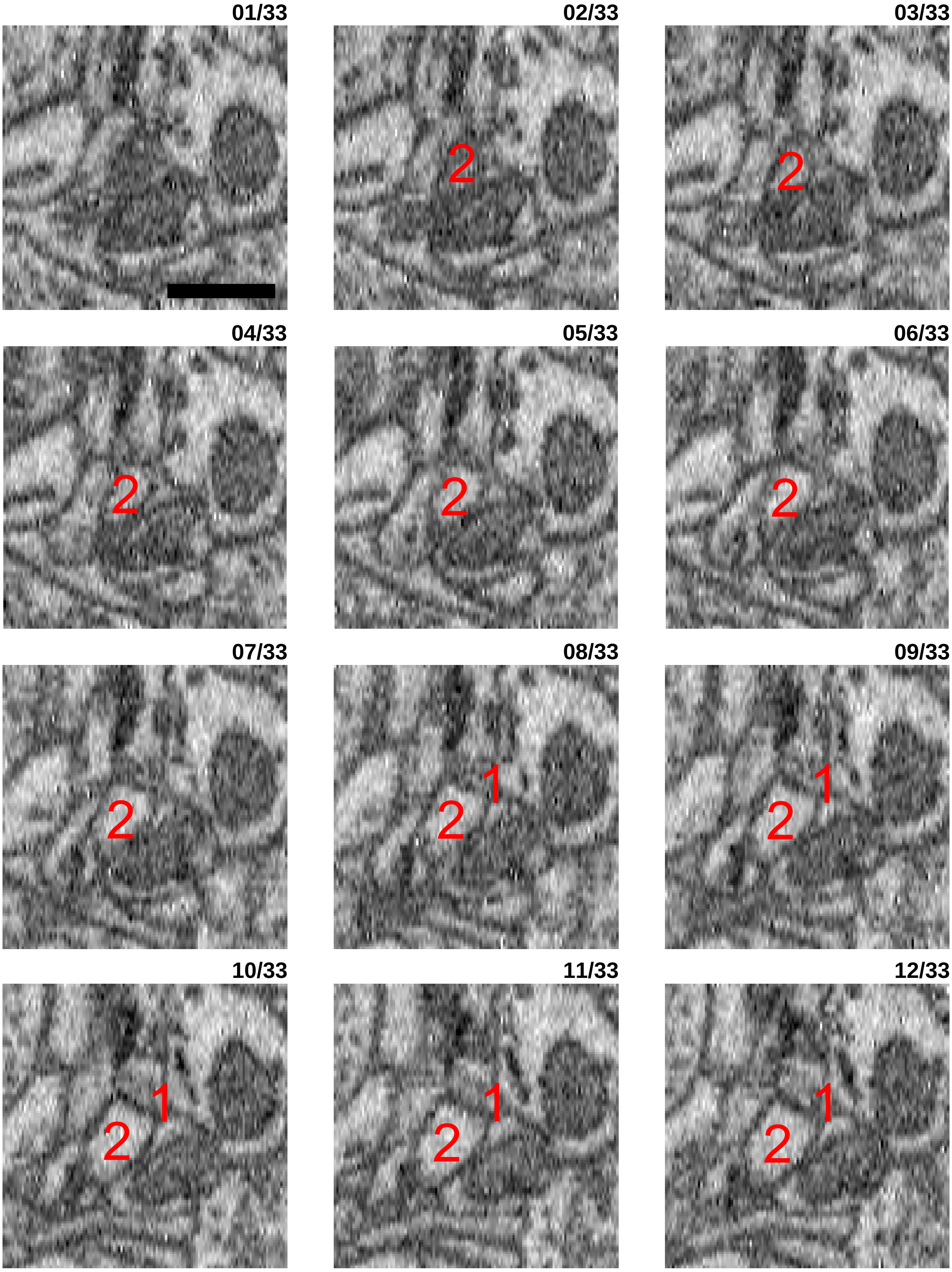

Potential synaptic contact between a very small axon (1) and a tiny spine head (2). Given that (2) is a small spine (see e.g. slices 26ff), the fact that an axon showing a certain intracellular accumulation of potential darkened appearance (slices 20ff) in direct proximity to the spine head was interpreted as a possible synaptic contact. Please note that this was the smallest of all contacts identified as synaptic by human experts and SynEM, and that this is in a size range where synapse detection agreement is substantially reduced for experts (see introduction).

True positive (TP) synapse 11 - cont (2)

xz orthogonal reslice

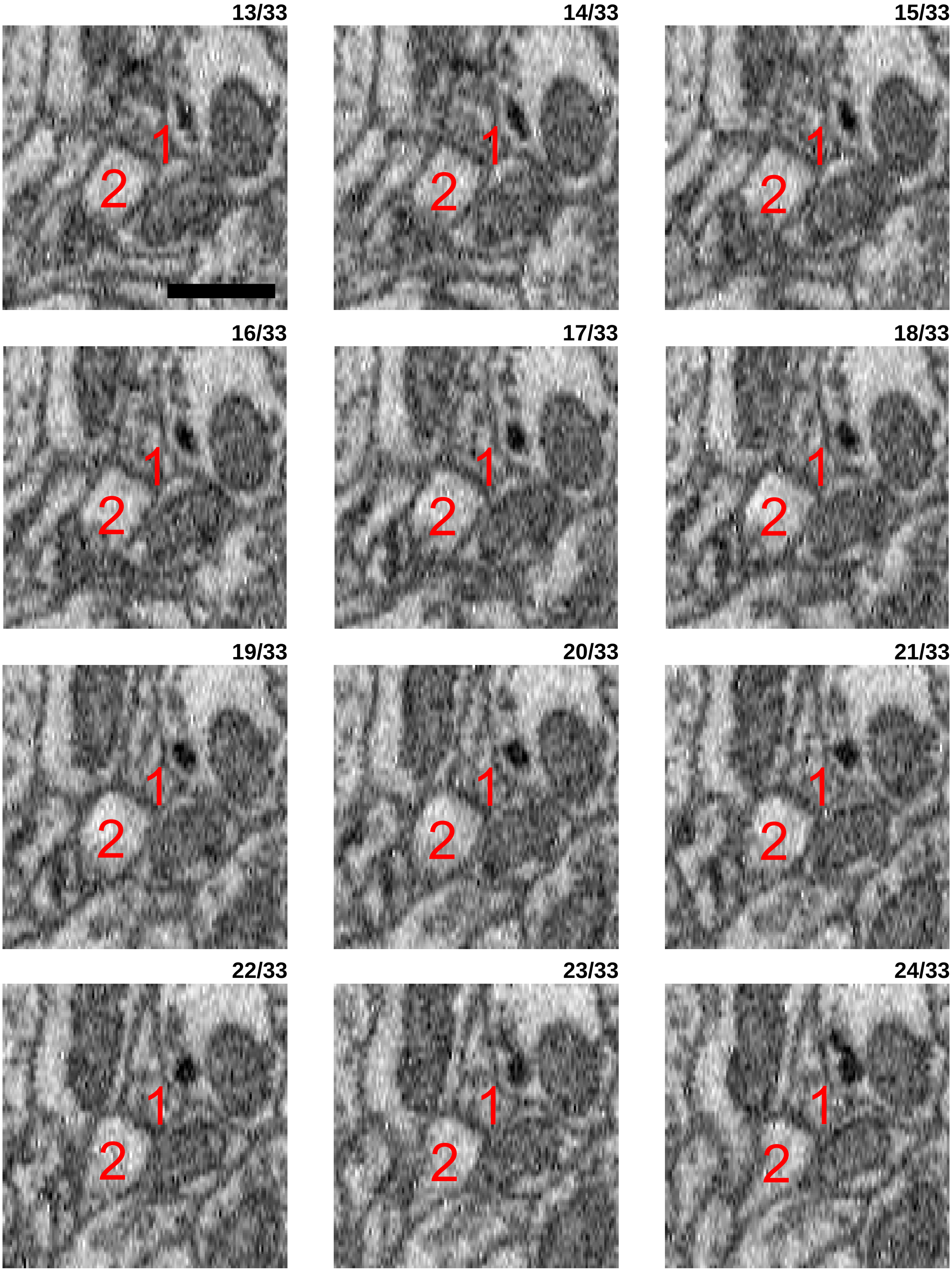

True positive (TP) synapse 11 - cont (3) xz orthogonal reslice

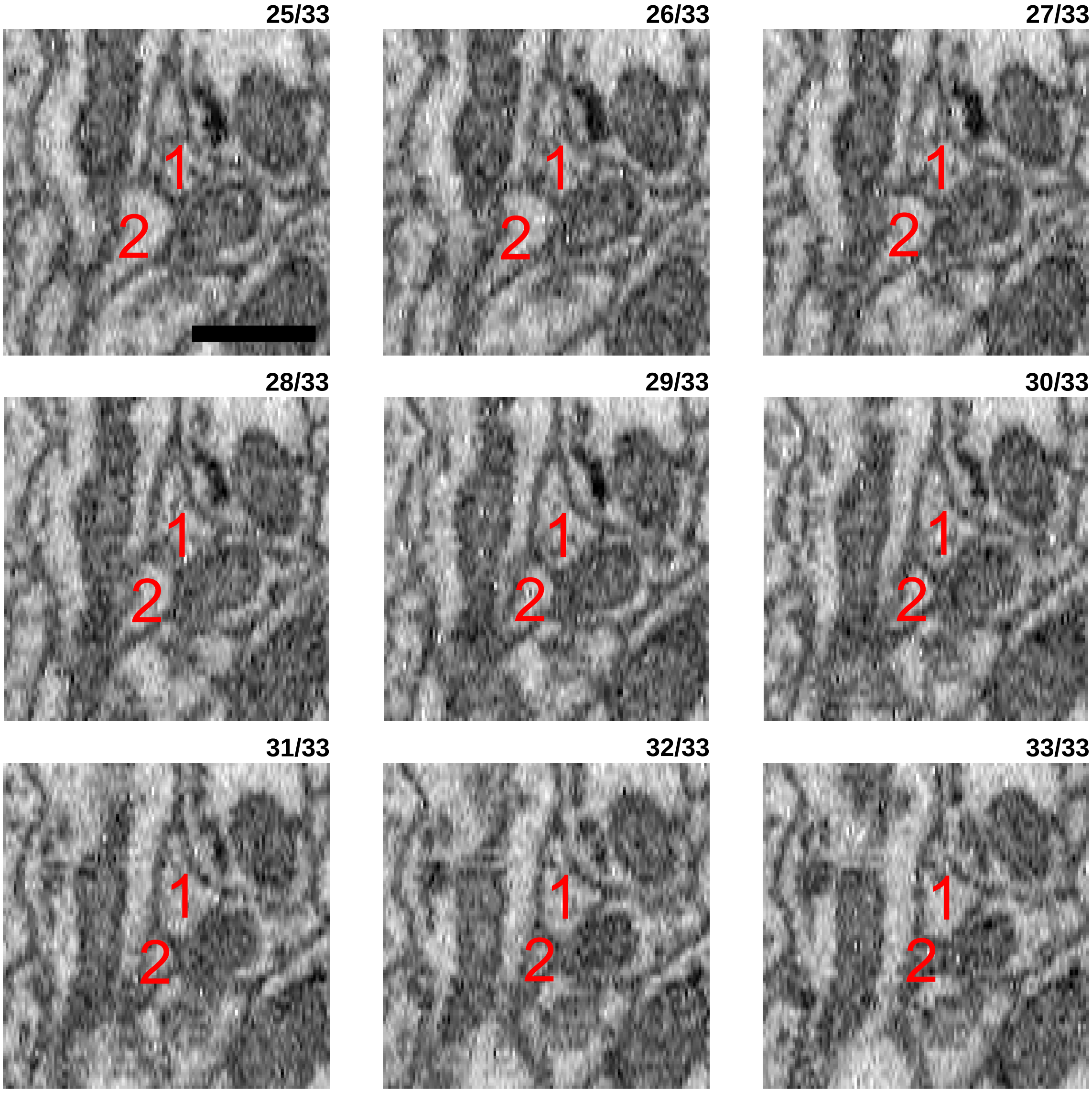

2 FN examples

(randomly selected)

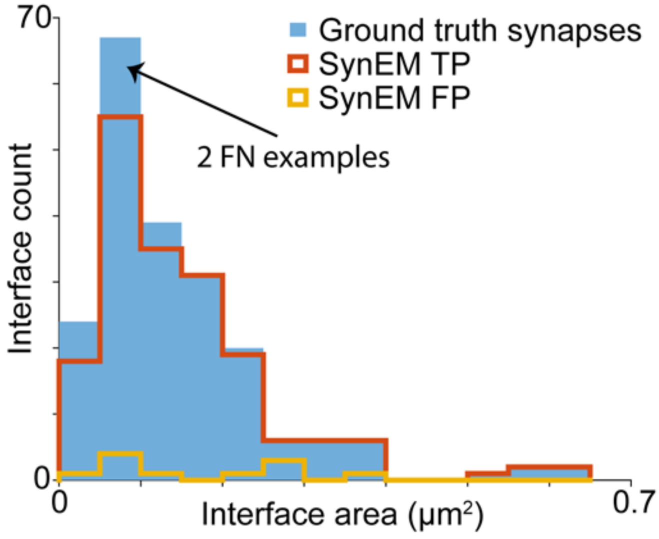

The following examples illustrate cases in which human experts detect a synapse, but SynEM did not (false negative detections, FN). Please note that these FNs are more frequent for small interfaces, where both human and SynEM synapse detection performance are lower.

False negative (FN) synapse 01 xy imaging plane

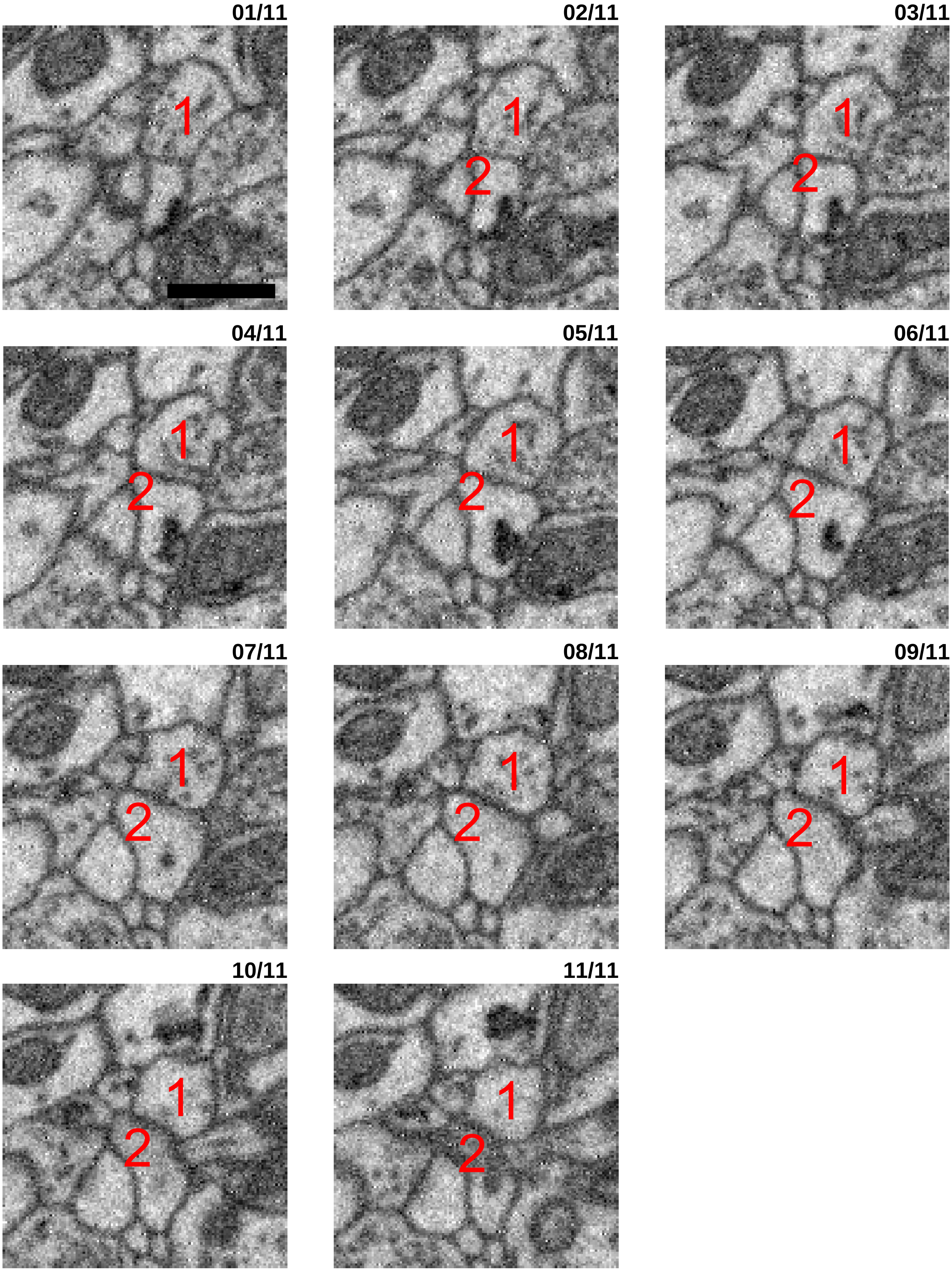

Potential synaptic interface between a very small axon (1) and a small dendrite (2). A very small set of potential vesicles can be discerned in slices 3-9, approaching the potential membrane, which has a certain darkening. This is clearly a questionable synapse for which human agreement is not high. If this is not considered a synapse, it would add to the TN classifications of SynEM, further improving SynEM recall.

False negative (FN) synapse 02 xy imaging plane

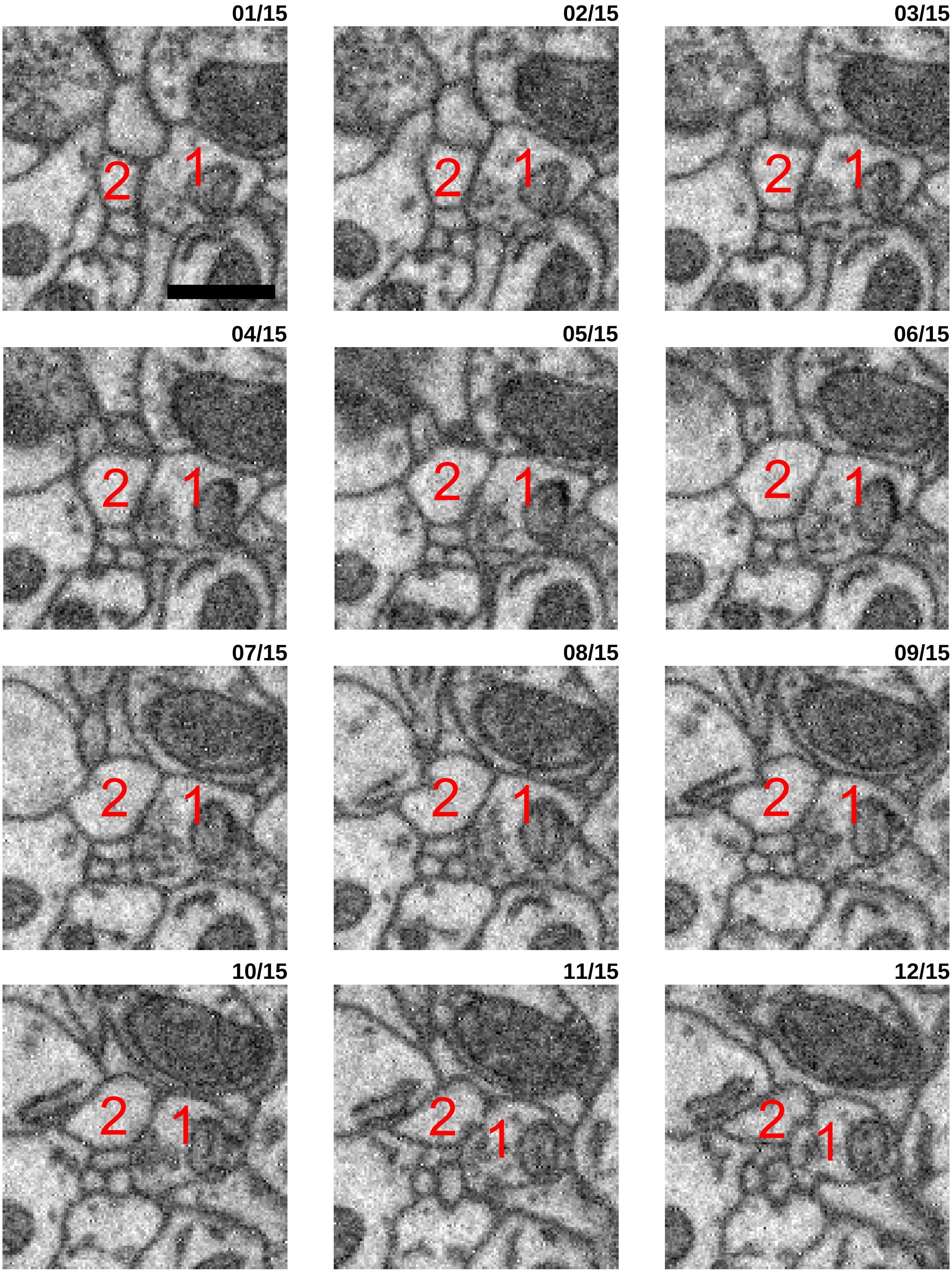

Synaptic interface between a small axon (1) and a small spine head (2) with only about a dozen vesicles attached to the membrane in slices 6ff and a certain darkening in slices 6, 7 and 8 is visible. It is a very small contact and the vesicle cloud is a little bit off center which may be contributing to the false negative detection.

False negative (FN) synapse 02 - cont (2)

xy imaging plane

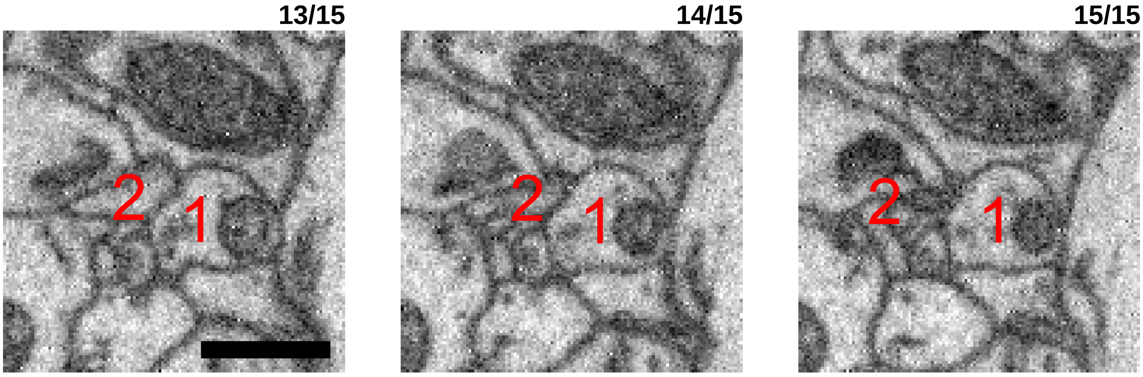

2 FP examples

(randomly selected)

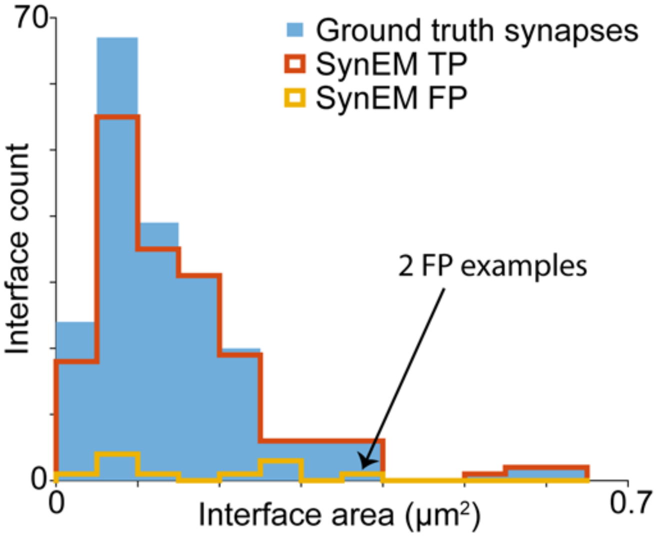

The following examples illustrate cases in which human experts did not detect a synapse, but SynEM did (false positive detections, FP).

False positive (FP) detection 01 xy imaging plane

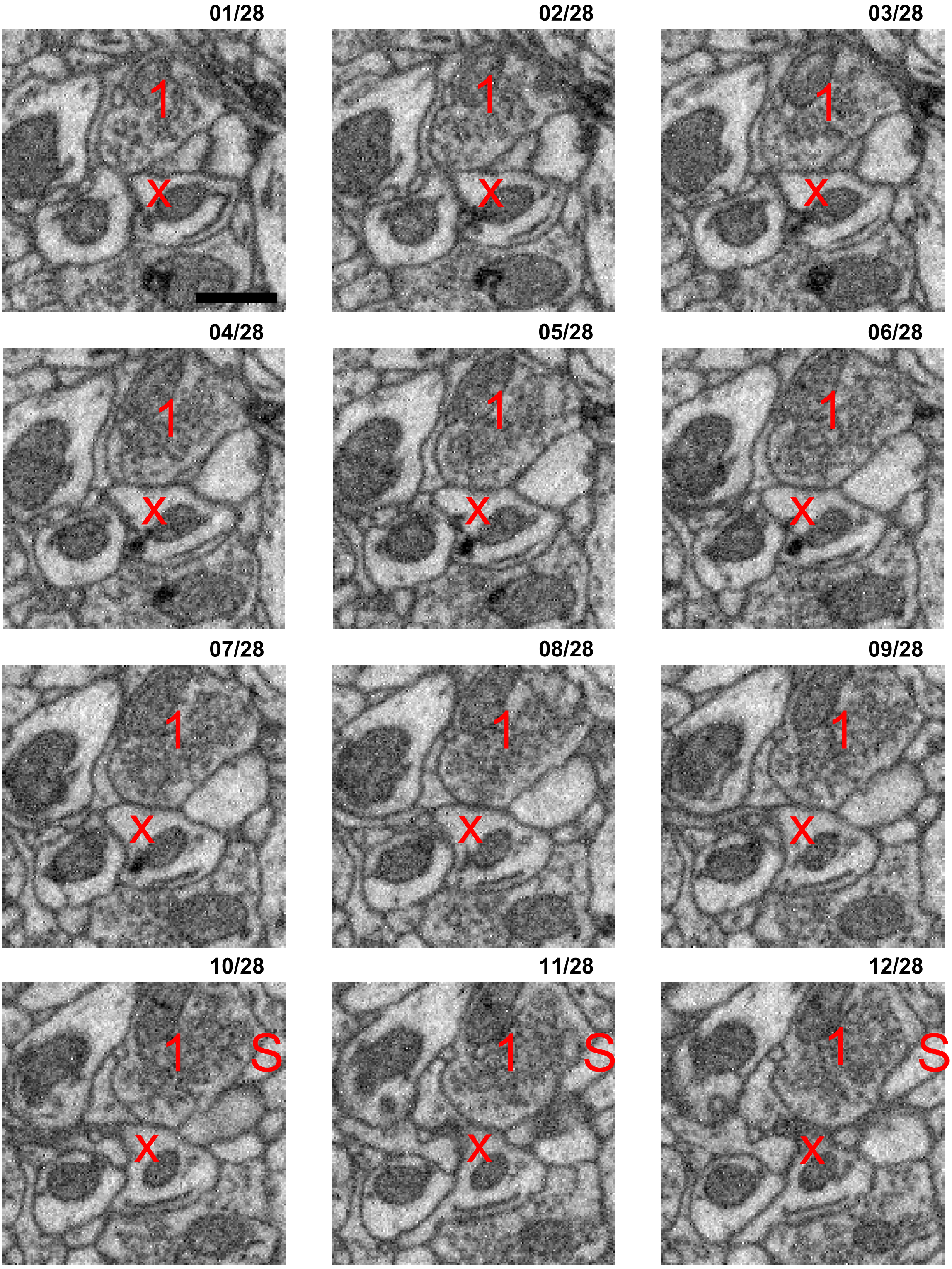

Contact between a large vesicle-filled presynaptic bouton (1) and a dendritic shaft (X). A small apposition of vesicles to the membrane can be imagined in slice 8 but is absent in all other slices and there is no broadening or darkening of the membrane. Furthermore, the bouton makes a synapse with a different spine head (S, slices 17ff). Therefore this was not considered a synapse.

False positive (FP) detection 01 - cont (2)

xy imaging plane

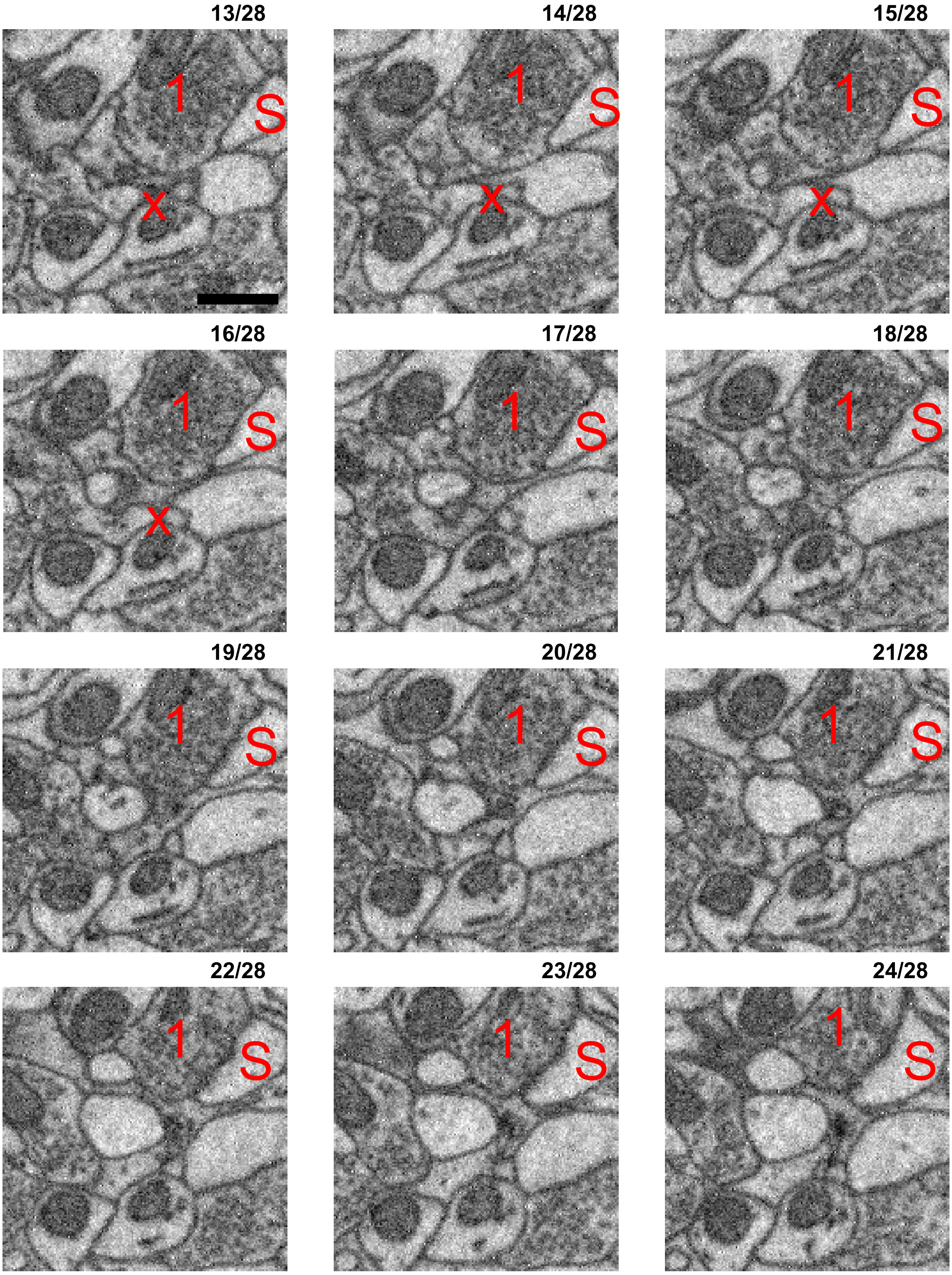

False positive (FP) detection 01 - cont (3) xy imaging plane

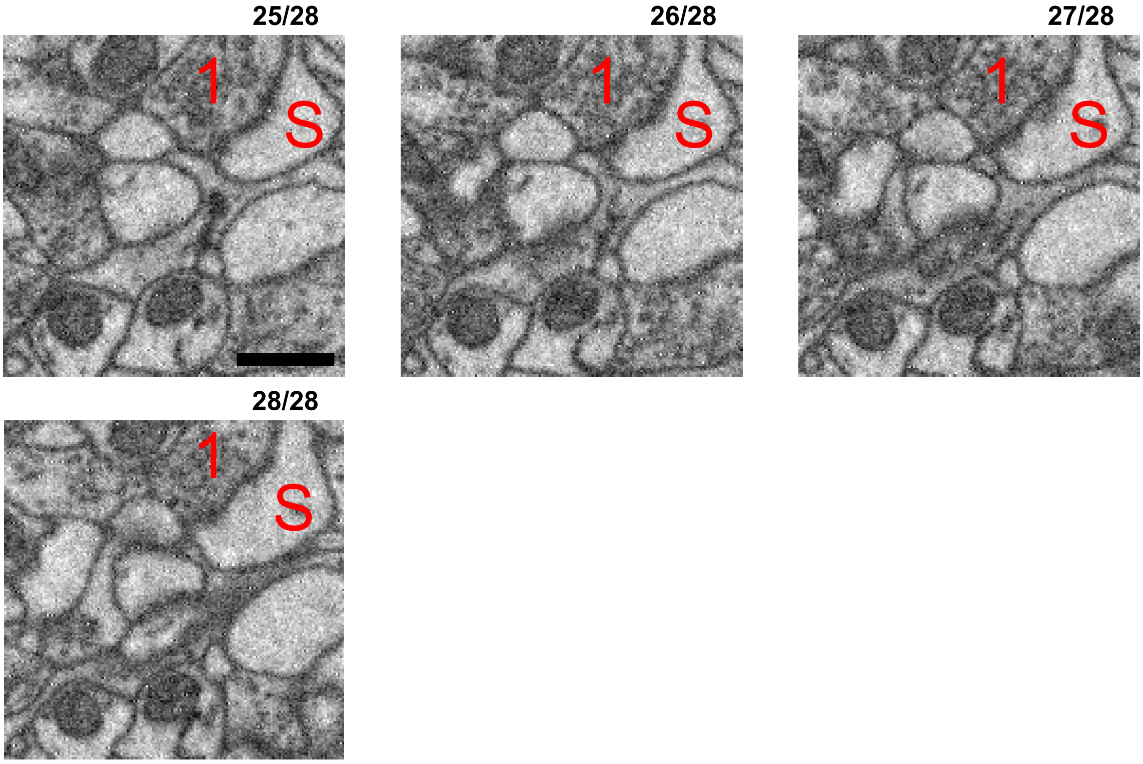

False positive (FP) detection 02 xy imaging plane

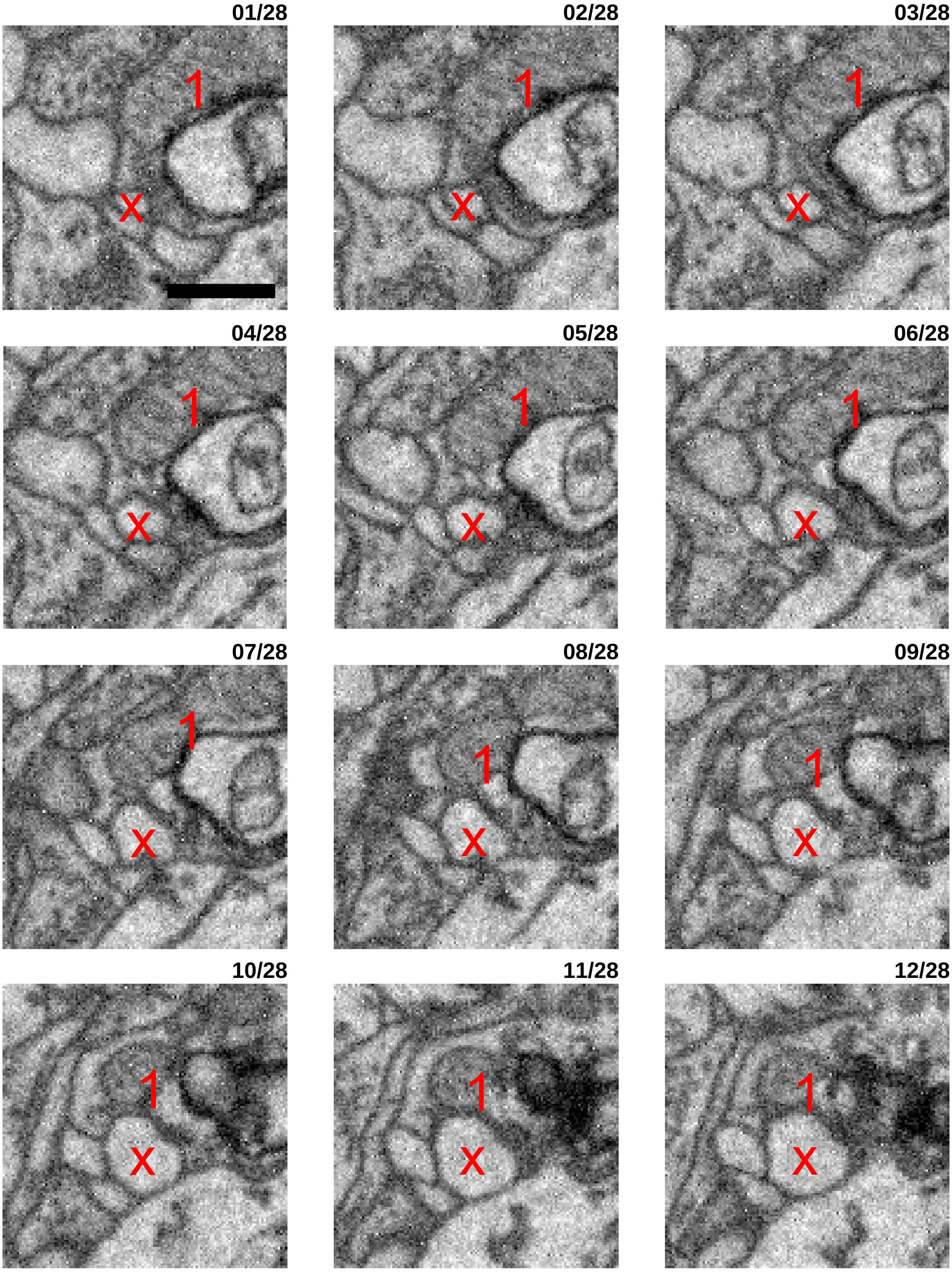

Interface between a mitochondrion (1) and likely spine head (X). Here the segmentation yielded an interface between an intracellular structure which is so close to the membrane (as seen in slices 10ff) that it imposed as a separate process. Since mitochondria can have tube-like shape in nerve tissue this is a rare but consistent phenomenon. This is not a synapse but was detected as one by the classifier.

False positive (FP) detection 02 - cont (2)   xy imaging plane

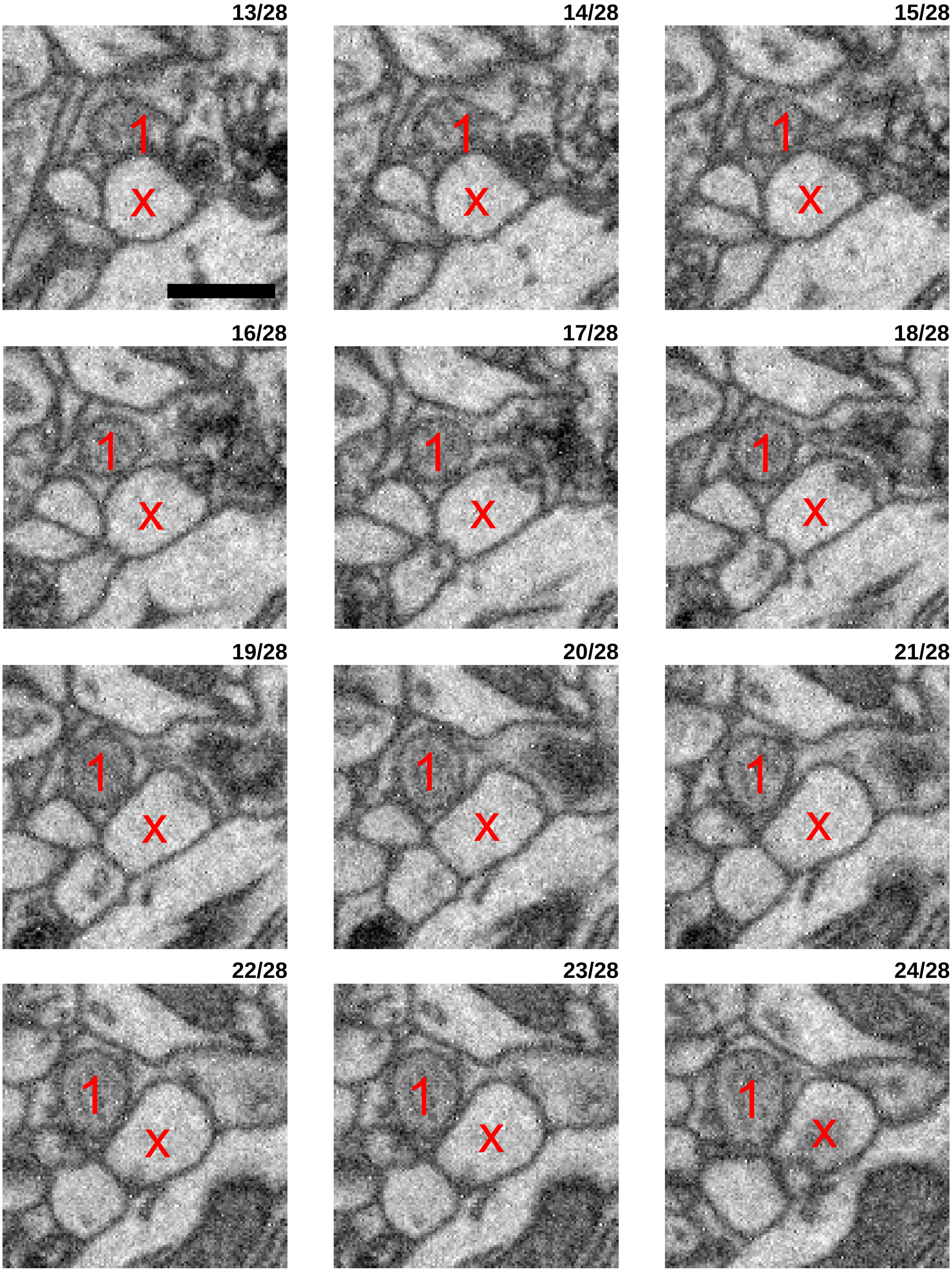

False positive (FP) detection 02 - cont (3) xy imaging plane

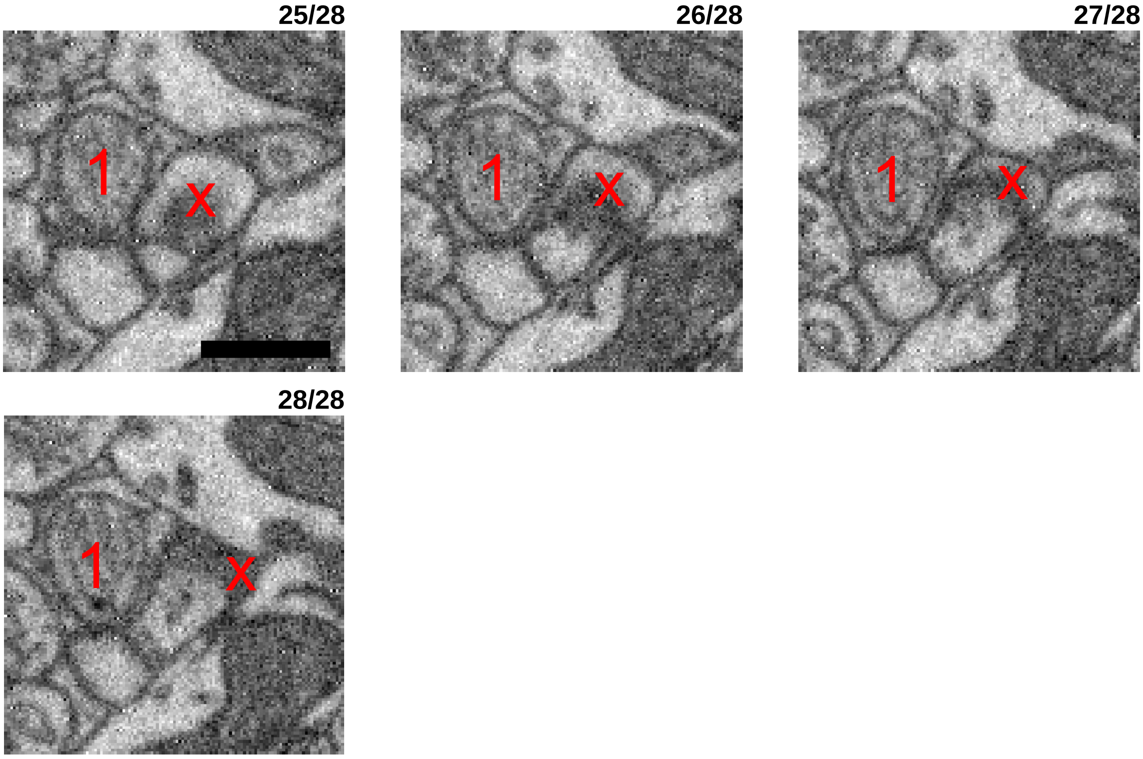

Cited references

Colonnier, M. (1968). Synaptic patterns on different cell types in the different laminae of the cat visual cortex. An electron microscope study. *Brain Res, 9*(2), 268-287.

Gray, E. G. (1959). Axo-somatic and axo-dendritic synapses of the cerebral cortex: an electron microscope study. *J Anat, 93*, 420-433.

Harris, K. M., Jensen, F. E., & Tsao, B. (1992). Three-dimensional structure of dendritic spines and synapses in rat hippocampus (CA1) at postnatal day 15 and adult ages: implications for the maturation of synaptic physiology and long-term potentiation. *J Neurosci, 12*(7), 2685-2705.

Palay, S. L. (1956). Synapses in the central nervous system. *J Biophys Biochem Cytol, 2*(4 Suppl), 193-202.
